# Transcriptome and metabolome profiling to elucidate the mechanism underlying the poor growth of *Streptococcus suis* serotype 2 after orphan response regulator CovR deletion

**DOI:** 10.3389/fvets.2023.1280161

**Published:** 2023-11-07

**Authors:** Bingbing Zong, Yong Xiao, Rui Li, Huanhuan Li, Peiyi Wang, Xiaopei Yang, Yanyan Zhang

**Affiliations:** ^1^Hubei Key Laboratory of Animal Nutrition and Feed Science, Engineering Research Center of Feed Protein Resources on Agricultural By-Products, Ministry of Education, Wuhan Polytechnic University, Wuhan, China; ^2^Wuhan Animal Disease Control Center, Wuhan, Hubei, China

**Keywords:** *Streptococcus suis* type 2, orphan response regulator CovR, Δ*covR*, metabolome profiling, transcriptome sequencing, poor growth, aminoacyl tRNA

## Abstract

The deletion of orphan response regulator CovR reduces the growth rate of *Streptococcus suis* serotype 2 (*S. suis* 2). In this study, metabolome and transcriptome profiling were performed to study the mechanisms underlying the poor growth of *S. suis* 2 caused by the deletion of orphan response regulator CovR. By comparing *S. suis* 2 (Δ*covR*) and *S. suis* 2 (SC19), 146 differentially accumulated metabolites (upregulated: 83 and downregulated: 63) and 141 differentially expressed genes (upregulated: 86 and downregulated: 55) were identified. Metabolome and functional annotation analysis revealed that the growth of Δ*covR* was inhibited by the imbalance aminoacyl tRNA biosynthesis (the low contents of L-lysine, L-aspartic acid, L-glutamine, and L-glutamic acid, and the high content of L-methionine). These results provide a new insight into the underlying poor growth of *S. suis* 2 caused by the deletion of orphan response regulator CovR. Metabolites and candidate genes regulated by the orphan response regulator CovR and involved in the growth of *S. suis* 2 were reported in this study.

## Introduction

*Streptococcus suis* (*S. suis*) is one of the main pathogenic bacteria on swine farms around the world (1). *S. suis* is also an emerging zoonotic pathogen that can cause a wide range of diseases (e.g., septicemia, endocarditis, arthritis, meningitis, and pneumonia) (2, 3). *S. suis* mainly causes death in 5- to 10-week-old post-weaned piglets, resulting in significant economic losses to the pig farming industry (4). According to the differences in capsular polysaccharide (CPS) antigenicity, 35 serotypes of *S. suis* strains were found and defined (5). Among these 35 serotypes, the *S. suis* 2 strain not only has the highest isolation rate but also poses a serious threat to the development of pig farming and human health (6).

Two-component systems (TCSs) play a crucial role in bacterial infection and response to changes in the external living environment, which have been identified in the vast majority of bacteria (7). The vast majority of TCSs in bacteria consist of a histidine kinase (HK) that receives stimulus signals and a cognate response regulator (RR) that transfers stimulus signals. Once HK receives external stimulus signals, a histidine residue located on the HK will be phosphorylated, and then the phosphoryl group is transferred to the aspartic acid residue on the RR. Subsequently, the phosphorylated RR will interact with the promoter areas of the target genes associated with the response to the environmental stimuli (8). TCSs can determine the survival and pathogenicity of bacteria by regulating various cellular processes, including competition, metabolism, antibiotic resistance, biofilm formation, pathogenicity, and stress response. Therefore, TCSs are viewed as attractive candidates for the development of new strategies for preventing bacterial infections (7, 9).

The CovR/S system consists of a sensor kinase gene (*covS*) and a RR gene (*covR*), which directly or indirectly regulate 15% of genes connected with bacterial pathogenesis in *Streptococcus* spp. (10–12). Due to the impact of CovR/S on a variety of critical virulence factors, it is considered the most essential TCS system for group A *Streptococcus* (GAS, *Streptococcus pyogenes*) (13). The CovR that can regulate the virulence of bacteria has been reported in many studies. In *Streptococcus agalactia*, for example, the Δ*covR* strain has stronger hemolytic activity, adherence ability to epithelial cells, and higher lethality in the neonate rat sepsis model (14). Previous studies reported that CovR can bind to the promoter areas of the majority of virulence factor genes in GAS (13). These reports suggested that CovR can negatively regulate bacterial virulence. In bacteria, protein tyrosine kinases are linked to pathogenicity and exopolysaccharide synthesis (15). In *Edwardsiella piscicida*, adenylosuccinate synthase has been reported to have an inhibition effect on the host's nuclear factor-kappa B (NF-κB) signaling pathway, which is a common and important immune signaling pathway (16). Hemolysin is the key virulence component of *S. suis* 2, which creates holes in the target cell membrane and activates the inflammasome NLRP3 in order to release cytoplasmic K^+^ efflux and cause Streptococcal toxic shock-like syndrome (17). Initiation factors IF1, IF2, and IF3 allow Shine-Dalgarno (SD) sequence-lead mRNAs initiate transcription (18). Ser/Thr kinase (STK) controls a number of cellular activities, including stress response, biofilm formation, membrane production, sporulation, metabolism, pathogenicity, and growth processes in bacteria. Recently, STK has been reported to be related to the virulence of *S. suis* (19). However, it is still unclear how the CovR/S system regulates genes and metabolites to promote the survivability of *S. suis* 2.

In recent years, transcriptomics and metabolomics have been extensively utilized to explain the survival and pathogenic mechanism of pathogenic bacteria. To reveal the mechanism by which the orphan RR factor CovR regulates the survivability of *S. suis* 2, liquid chromatography-tandem mass spectrometry (LC-MS) was employed to dig for the differentially expressed metabolites between *S. suis* 2 (wild type) and Δ*covR* (mutant) strains. Furthermore, the assessment of the differentially expressed genes (DEGs) was carried out using RNA-Seq and qRT-PCR. This study will not only reveal the genes and metabolites that are regulated by the orphan RR factor CovR involved in the survivability of *S. suis* 2 but also provide some potential targets for preventing and controlling *S. suis* 2 infections.

## Materials and methods

### Bacterial strains and growth condition

The *S. suis* 2 SC19 strain was donated by Prof. Chen Tan of Huazhong Agricultural University. SC19 is plated on Tryptose Soya agar (TSA) or cultured in Tryptose Soya broth (TSB) (Difco Laboratories, Detroit, MI, USA) with 10% (v/v) newborn bovine serum (Sijiqing Biological Engineering Materials Co., Ltd., Hangzhou, China) at 37°C. Δ*covR* mutant and complemented CΔ*covR* strains are stored in our laboratory and cultured as described above (20).

### Growth characteristics

The growth characteristics of wild-type SC19, Δ*covR* mutant, and complemented CΔ*covR* strains were determined by transferring overnight bacterial cultures into fresh preheated TSB supplemented with 10% newborn bovine serum at a ratio of 1:1000. The bacteria were grown at 37°C, accompanied by shaking at 180 rpm for 12 h, and OD_600_ was determined by an Automatic Microbial Growth Curve Analyzer (MGC-200, Ningbo Scientz Biotechnology Co., Ltd., Ningbo, China) for every hour. One-way ANOVA test was used to verify the significance of growth characteristics of SC19, Δc*ovR*, and CΔ*covR* for every hour.

### RNA extraction, library construction, and sequencing

The total RNA was extracted using the TRIzol reagent (Takara, Dalian, China) from SC19 (*n* = 3) and Δ*covR* (*n* = 3) as described by Rio et al. (21), and the genomic DNA in the total RNA was removed by using DNase I (Takara). A total of 2 μg of RNA was used to build the RNA-seq transcriptome library by using the TruSeq^TM^ RNA sample preparation Kit (Illumina, San Diego, CA). Briefly, the Ribo-Zero Magnetic Kit (epicenter) was used to exhaust ribosomal RNA (rRNA), and then the fragmented mRNA was broken into short fragments (200 bp) by using a fragmentation buffer. The mRNA fragment was performed by RT-PCR using the SuperScript Double-Stranded cDNA Synthesis Kit (Invitrogen, CA) with random hexamer primers (Illumina). The dTTP was replaced with dUTP when the second strand was synthesized. Then, after end-repair and phosphorylation, the synthesized cDNA was treated with “A” base addition according to the protocol of Illumina's introduction. The UNG enzyme was used to recognize and degrade those second-strand cDNA with dUTP. The library was constructed using 200-bp cDNA target fragments of 2% low-range ultra-agarose. Subsequently, these fragments were amplified by performing 15 PCR cycles with Phusion DNA polymerase and quantified using TBS380. Finally, the paired-end RNA-seq sequencing library was constructed using Illumina Novaseq (2 × 150 bp read length).

### Transcriptome data analysis

The raw data collected from Illumina Hiseq sequencing were converted into raw data using base calling, and the results were saved in FASTQ file format. For mapping assembly sequences and distinct genes to reference sequences (GenBank: CP020863.1), Bowtie with default parameters (http://bowtiebio.sourceforge.net/index.shtml) was employed. FPKM (fragments per kilobase million) was used to quantify the quantity of genes and transcripts (http://deWeylab.biostat.wisc.edu/rsem/, https://ccb.jhu.edu/software/stringtie/). The transcriptome data were analyzed by the cloud platform of Shanghai Majorbio Bio-Pharm Technology Co., Ltd. (Shanghai, China). Additionally, the DEGs between Δ*covR* and *S. suis* 2 strains were detected using the DESeq2 software. To control the probability of error with multiple test corrections (Benjamini/Hochberg, BH), the *p*-values were adjusted. DEGs were identified as their fold change ≥1.2 or ≤ 0.8 and adjusted *p*-values < 0.05. The GO analysis for DEGs was performed using the Goatools software, while the KEGG analysis was performed using R script.

### Extraction of metabolites from bacteria and quality control measurement

Overnight, SC19 (*n* = 4) and Δ*covR* (*n* = 4) cultures were diluted at a 1:1000 ratio into TSB supplemented with 10% newborn bovine serum, and the mixture was then incubated to the logarithm phase. A total of 100 ml of the bacterium was transferred to a clean tube and centrifuged at 12,000*g* at 4°C for 10 min, and then the supernatant was removed. The metabolites were extracted from the bacterium (SC19 and ΔcovR) by a 400-μl isovolumetric methanol and acetonitrile solution and then sonicated by an ultrasonic cleaner (SBL-10TD, Ningbo Xinzhi Biotechnology Co., Ltd.) at 40 kHz for 30 min at 5°C. After being placed at −20°C for 30 min, the mixture was centrifuged at 13,000*g* at 4°C for 15 min. The supernatant was carefully transferred to a new microtube and evaporated to dry under a gentle stream of nitrogen. Then, the samples were resuspended with 100 μl of loading isovolumetric acetonitrile and water solution by short sonication using an ultrasonic cleaner (Ningbo Scientz Biotechnology Co., Ltd.) in a 5°C water bath. The extracted metabolites were centrifuged at 13,000*g* for 15 min at 4°C and then UHPLC-MS/MS analysis was performed. The same volumes of all samples were mixed to be used as a pooled quality control.

### UHPLC-MS/MS analysis

The LC-MS System (UHPLC-Q Exactive HF-X System, Thermo Fisher Scientific, Shanghai, China) was used to analyze the extracted metabolites. First, the sample was injected into a Waters HSS T3 column (100 mm × 2.1 mm i.d., 1.8 μm), which was followed by mass spectrometry detection. The mobile phases consisted of two solvents: solvent A, which consisted of 95% 0.1% formic acid and 5% acetonitrile, and solvent B, which consisted of 47.5% 0.1% formic acid in acetonitrile with 47.5% isopropanol and 5% water. The solvent gradient employed the following conditions: First, the gradient changed from 0 B to 24.5% B at a flow rate of 0.4 ml/min for 3.5 min; then, the gradient changed from 24.5 B to 65% B for 1.5 min; the gradient changed from 65 B to 100% B for 0.5 min; the gradient remained at 100% B for 1.9 min with the flow rate being increased from 0.4 to 0.6 ml/min; the gradient changed from 100 B to 51.5% B for 0.2 min; the gradient changed from 51.5 B to 0% B for 0.2 min at a flow rate of 0.6 to 0.5 ml/min; the gradient remained at 0% B for 0.2 min at a flow rate of 0.5 to 0.4 ml/min; the system equilibration was performed as the gradient remained at 0% B for 1 min at a flow rate of 0.4 ml/min. The sample injection volume was 2 μl at a flow rate of 0.4 ml/min. The column temperature was maintained at 40°C throughout the analysis, and the samples were stored at 4°C. The mass spectrometric data were collected using continuous scanning in either positive or negative ion modes. Data acquisition was performed in the data-dependent acquisition mode. The full MS resolution was set at 60,000, and the MS/MS resolution was set at 7,500. The heater temperature and capillary temperature were set at 425°C and 325°C, respectively. The sheath gas flow rate was set at 50 arb and the aux gas was set at 13 arb. The ion-spray voltage floating was −3,500 V in negative mode and 3,500 V in positive mode. Normalized collision energy for MS/MS was set at 20-40-60 V rolling. The detection range for mass spectrometry was from 70 to 1,050 m/z.

### Metabolite data and differential metabolites analysis

A three-dimensional data matrix that included sample information, metabolite name, and mass spectral response intensity in CSV format was obtained by analyzing metabolite data with Progenesis QI (Waters Corporation, Milford, USA). Internal standard peaks, as well as any known false positive peaks that include noise, column bleed, and derivatized reagent peaks, were removed from the data matrix, which was then de-redundant and peak pooled. The metabolic mass spectra features were recognized by accurate mass, MS/MS fragment spectra, and isotope ratio differences were searched in reliable biochemical databases such as the Human Metabolome Database (HMDB) (http://www.hmdb.ca/) and the METLIN database (https://metlin.scripps.edu/).

After searching in the database, these data were uploaded to the Majorbio cloud platform (https://cloud.majorbio.com) for analysis. In each set of samples, at least 80% of the metabolic features detected were retained. The metabolic levels in specific samples that fell below the lower limit of quantitation were imputed with minimum metabolite values, and each metabolic feature was normalized by sum. The response intensity of the sample mass spectrum peaks was normalized using the sum normalization method to obtain the normalized data matrix. Those QC samples with variables that had a relative standard deviation >30% were removed, and a logarithmic transformation (log10) was applied to the final data matrix for subsequent analysis.

Next, principal component analysis (PCA) and orthogonal least squares partial discriminant analysis (OPLS-DA) were conducted on the preprocessed data array using the R package (Version 1.6.2). The stability of the model was evaluated through seven cycles of cross-validation. To identify significantly different metabolites, VIP values obtained from the OPLS-DA model and Student's *t*-test were used, with VIP >1 and *p* < 0.05 indicating significance. Metabolic pathway annotation was carried out using the KEGG data (https://www.kegg.jp/kegg/pathway.html) to determine the pathways with which the differential metabolites were associated. Pathway enrichment analysis was performed using the Python package scipy.stats, and Fisher's exact test was used to identify the most relevant biological pathways to the experimental treatments. The raw data were finally uploaded to MetaboLights, where they were given the unique identifier MTBLS8495 (www.ebi.ac.uk/metabolights/MTBLS8495).

### Quantitative real-time polymerase chain reaction

In this study, the accuracy of RNA-seq data was validated by performing qRT-PCR on the selected DEGs. Among them, B9H01_RS09035 is glutamine ABC transporter substrate-binding protein; B9H01_RS06585 is glutamine ABC transporter permease; B9H01_RS06580 is glutamine ABC transporter permease; B9H01_RS05040 is peptide ABC transporter ATP-binding protein; B9H01_RS05035 is amino acid ABC transporter permease; B9H01_RS09790 is ABC transporter ATP-binding protein; and B9H01_RS01490, B9H01_RS06570, and B9H01_RS09040 are ABC transporters. To begin, RNA extraction was followed as described in the preceding part of the text, and 1 μg of RNA was reserved for transcription using a PrimeScript^®^ RT reagent Kit and gDNA Eraser (Takara, Dalian, China). Next, a 7500 Fast Real-Time PCR System (Applied Biosystems, Foster City, CA, USA) was used to perform the PCR reaction using Taq Pro Universal SYBR qPCR Master Mix (Q712, Vazyme, Nanjing, China). The 16S RNA gene served as the control gene, and all mRNA transcription levels were reported as 2^−ΔΔ*Ct*^. The primers in Table 1 were designed according to the genomic sequence of SC19 and used for qRT-PCR.

**Table 1:** Primers used in this study.

**Gene**	**Forward (5^′^-3^′^)**	**Reverse (5^′^-3^′^)**
B9H01_RS09035	GGGGCTAACAAACAGACT	AAGACACCGATGAGGAGA
B9H01_RS06585	CCGAGATTATCCGTGGTG	GCCTCAAAGTAGCGTCCC
B9H01_RS06580	TGCGTCGGACTCTATCAC	TGGAAGAATGGTCGGAAT
B9H01_RS05040	CCTTCCGTTCAAATGTCG	CGTTGCTTCTGACCACCT
B9H01_RS05035	CGTTATCGCTACCACAAA	ACAGCACCAGCAGAAAGG
B9H01_RS01490	TTGGCAGGACATCTATTA	AGGAAACGAAACATTACTC
B9H01_RS09790	AAATCGGGCAGGCTTACT	CCTTACGCATACGGTTGG
B9H01_RS06570	TTTTGGGAATCAATAAGGC	CGGATAGCATAGCAGGGT
B9H01_RS09040	CTCAATCATCGGTTCATC	CACGGTAAGTCGTCAAAT
16S rRNA	ACTTGAGTGCAGAAGGGGAGAG	GCGTCAGTTACAGACCAGAGAGC

### Statistical analyses

The data in this study were analyzed using SPSS (Version 17.0, SPSS Inc., Chicago, IL, USA) and GraphPad Prism 8. Unpaired *t*-tests between two groups or one-way ANOVA among multiple groups were used to obtain *p*-values (^*^p < 0.05, ^**^p < 0.01, ^***^p < 0.001; NS, not significant).

## Results

### The effects of covR deletion on the growth characteristics of SC19, Δ*covR*, and cΔ*covR* strains

**Figure 1:**
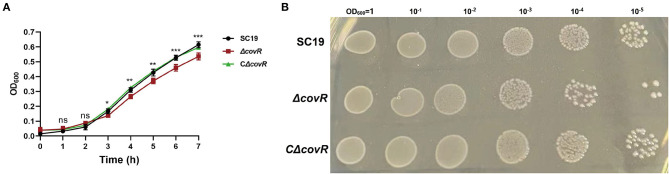
The growth characteristics of SC19, Δ*covR*, and complemented CΔ*covR* strains. **(A)** The growth curves of SC19, Δ*covR*, and complemented CΔ*covR* strains. **(B)** The clones of SC19, Δ*covR*, and complemented CΔ*covR* strains. Compare SC19 and Δ*covR*; ns, no significance; *p < 0.5, **p < 0.01, ***p < 0.001.

As shown in Figure 1, the growth of Δ*covR* was significantly slower compared to that of SC19 and the complemented CΔ*covR* strains after 3 h.

### Metabolome profiling

For the purpose of determining the poor growth of mutant Δ*covR*, metabolome profiling via untargeted LC-MS was performed on SC19 and mutant Δ*covR*. The metabolites identified from LC-MS were presented in Supplementary Table S1. There was a total of 514 metabolites identified, which were classified into 11 categories. Supplementary Figure S1 provides the PCA of SC19, Δ*covR*, and QC samples, which present significant separation. The coloration of each sample was analyzed using metabolite concentration data and shown in Supplementary Figure S1, which suggests that different samples were distinguished significantly. This means that the metabolome data were highly reliable. These results also suggested that SC19 and Δ*covR* samples had substantially different metabolite profiles.

### DAMs between SC19 and mutant Δ*covR* strains

**Figure 2:**
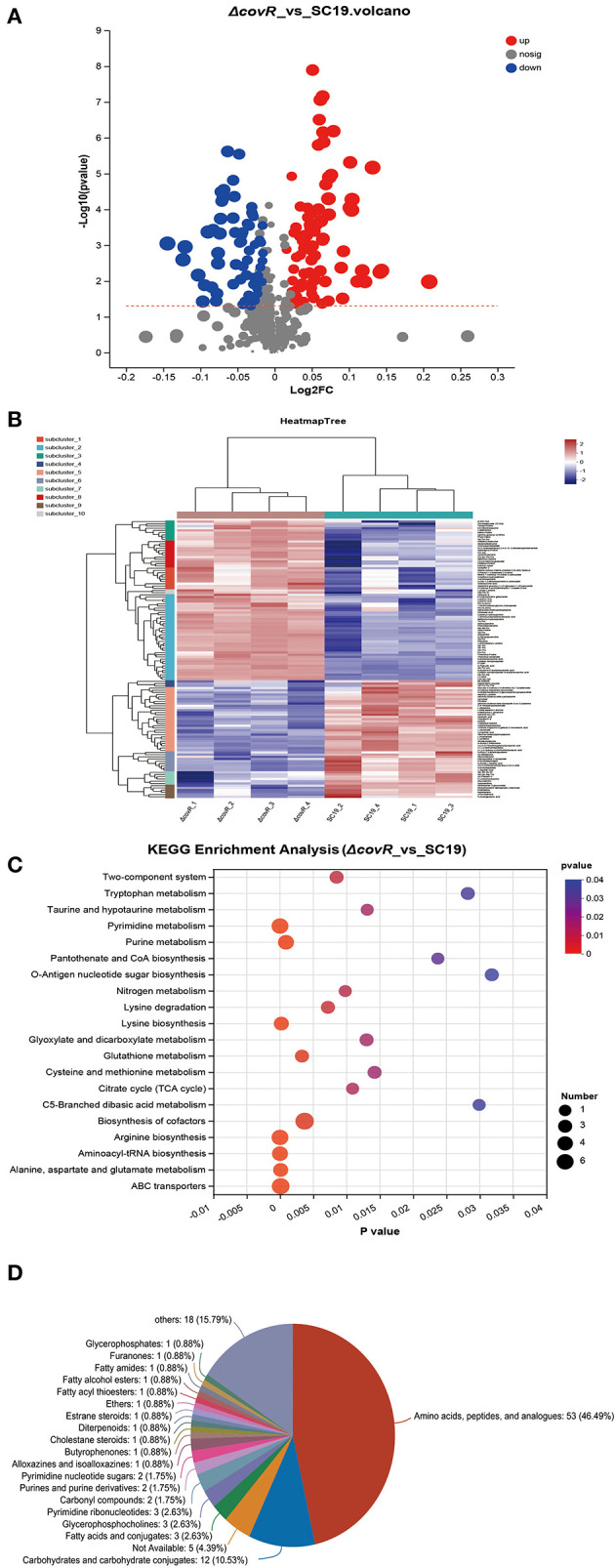
Differentially accumulated metabolites between SC19 and mutant Δ*covR* strains. **(A)** Volcano plots displayed DAMs that showed significant change (upregulated and downregulated) or no significant change between SC19 and mutant Δ*covR* strains. **(B)** The heat map of mutant Δ*covR* and *two* SC19 strains. **(C)** KEGG enrichment analysis of DAMs. **(D)** Pie chart depicting the categories of DAMs.

The uniquely mapped read ratio of all samples was higher than 96%, which means the reference sequence was reliable. To acquire Differentially accumulated metabolites (DAMs) between SC19 and mutant Δ*covR* strains, parameters such as *p*-values <0.05, |Log2 Fold Change| >1, and variable importance in the projection (VIP) >1 were used to analyze all metabolite data. In total, 146 DAMs (83 were upregulated and 63 were downregulated) were acquired (Figures 2A, B, Supplementary Table S2). The results of the Kyoto Encyclopedia of Genes and Genomes (KEGG, Figure 2C) analysis showed that arginine biosynthesis (map00220), pyrimidine metabolism (map00240), aminoacyl-tRNA biosynthesis (map00970), alanine, aspartate and glutamate metabolism (map00250), ABC transporters (map02010), and lysine biosynthesis (map00300) were significantly enriched. Further analysis results showed that DMAs in these enrichment pathways were likely involved in aminoacyl-tRNA biosynthesis, energy metabolism, and amino acid biosynthesis and metabolism (Table 2). In addition, the 146 DMAs could be categorized into >10 classes (Figure 2D). These analysis results suggest that DAMs involved in aminoacyl-tRNA biosynthesis, energy metabolism, and amino acid biosynthesis and metabolism may play a key role in slowing down the growth rate of the mutant Δ*covR* strain.

**Table 2:** The list of 15 DAMs identified between *S. suis* 2 SC19 and mutant Δ*covR* strains.

	**Metabolite**	**Formula**	**Peak area**		**VIP**	**FC (Δ*covR*/SC19)**	***p-*value**	**Type**
			Δ***covR***	**SC19**				
Aminoacyl-tRNA biosynthesis (peptides)	L-Aspartic acid	C4H7NO4	4.666 ± 0.048	5.072 ± 0.130	2.566141	0.919771	0.001118	Down
	L-Methionine	C5H11NO2S	3.878 ± 0.026	3.637 ± 0.082	1.965388	1.066263	0.001472	Up
	L-Lysine	C6H14N2O2	4.474 ± 0.048	4.493 ± 0.042	0.271064	0.99555	0.5672	Down
	L-Glutamate	C5H9NO4	5.949 ± 0.015	6.032 ± 0.021	1.171411	0.986077	0.000725	Down
	L-Glutamine	C5H10N2O3	4.344 ± 0.024	4.456 ± 0.037	1.297029	0.974871	0.002603	Down
Amino acid biosynthesis and metabolism	1-Aminocyclopropanecarboxylic acid	C4H7NO2	6.041 ± 0.009	5.933 ± 0.029	1.344261	1.0182	0.000447	Up
	Citrulline	C6H13N3O3	6.063 ± 0.015	6.132 ± 0.009	1.071567	0.988748	0.000282	Down
	L-Ornithine	C5H12N2O2	4.701 ± 0.008	4.912 ± 0.022	1.904153	0.957053	2.41E-06	Down
	N-acetylaspartate	C6H9NO5	4.426 ± 0.057	4.891 ± 0.141	2.755653	0.904742	0.000897	Down
	Allysine	C6H11NO3	5.775 ± 0.009	5.580 ± 0.043	1.81366	1.03494	0.000127	Up
	N-Acetyl-L-aspartic acid	C6H9NO5	5.640 ± 0.048	6.002 ± 0.091	2.444165	0.939687	0.000427	Down
	N-Acetylglutamic acid	C7H11NO5	4.726 ± 0.068	5.054 ± 0.236	2.083366	0.935101	0.0373	Down
Energy	Oxalosuccinic acid	C6H6O7	4.227 ± 0.049	4.139 ± 0.034	1.082973	1.021256	0.02676	Up
	Acetyl-CoA	C23H38N7O17P3S	4.885 ± 0.059	4.585 ± 0.205	1.97304	1.065416	0.03078	Up
	D-4'-Phosphopantothenate	C9H18NO8P	5.545 ± 0.091	5.847 ± 0.088	2.132044	0.948521	0.003222	Down

### Transcriptome profiles between SC19 and mutant Δ*covR* strains

**Figure 3:**
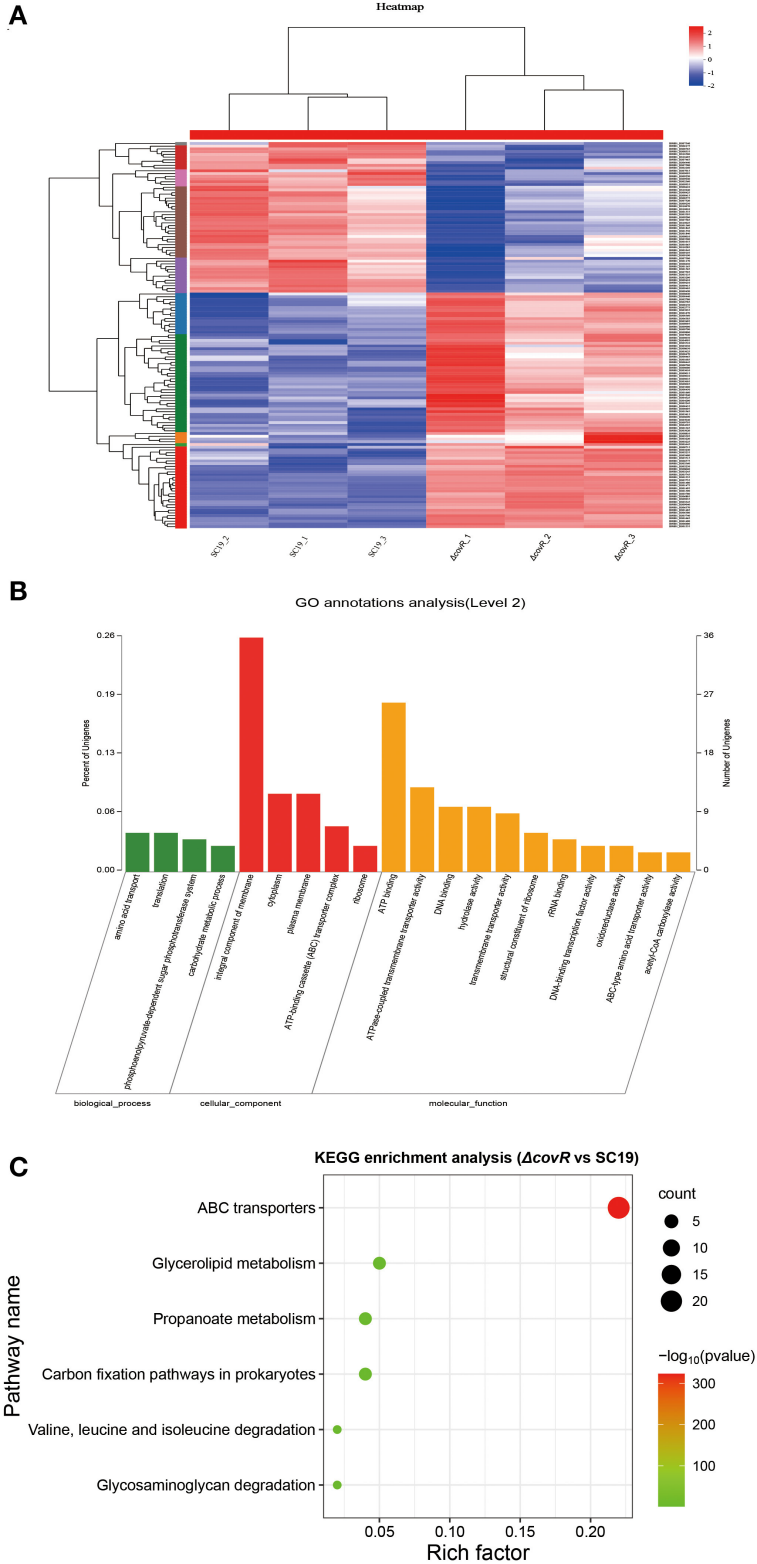
DEGs between mutant Δ*covR* and SC19 strains. **(A)** The heat map of mutant Δ*covR* and SC19 strains. **(B)** Go annotation analysis. **(C)** The KEGG enrichment pathways of 141 DEGs.

The DEGs between SC19 and mutant Δ*covR* strains were analyzed. There were 145,666,522 (SC19, 72,699,732; mutant Δ*covR*, 72,966,790) total reads generated by RNA-seq. A total of 141,860,342 (SC19, 70,781,282; mutant Δ*covR*, 71,079,060) clean reads were obtained by eliminating the low-quality reads. A total of 141,860,342 (SC19, 69,990,276; mutant Δ*covR*, 70,298,114) mapped reads were collected by aligning the high-quality reads to the reference genome (two SC19 chromosomes, complete genome, GenBank: CP020863.1). The correlation analysis showed significantly different relationships between Δ*covR* and SC19 strains (Figure 3A).

### DEGs between 2 SC19 and mutant Δ*covR* strains

To explore the candidate genes responsible for poor growth of Δ*covR* strains, DEGs (|Log2 Fold Change| >1.2 or <0.8) were identified between SC19 and mutant Δ*covR* strains. A total of 141 DEGs (86 were upregulated and 55 were downregulated) were identified. The GO annotation analysis result showed that the 141 DEGs were classified into three categories (Figure 3B), among which the integral component of membrane (36, 25.5%) was the largest group; the second largest group was ATP binding (26, 18.4%); only a few were related to metal ion binding (3, 2.1%) and iron-sulfur cluster assembly (3, 2.1%). As shown in Figure 3C, the enrichment pathways in KEGG terms were ABC transporters, glycerolipid metabolism, propanoate metabolism, carbon fixation pathways in prokaryotes, valine, leucine, and isoleucine degradation, and glycosaminoglycan degradation (Supplementary Table S3).

### The correlation analysis of DAMs and DEGs

**Figure 4:**
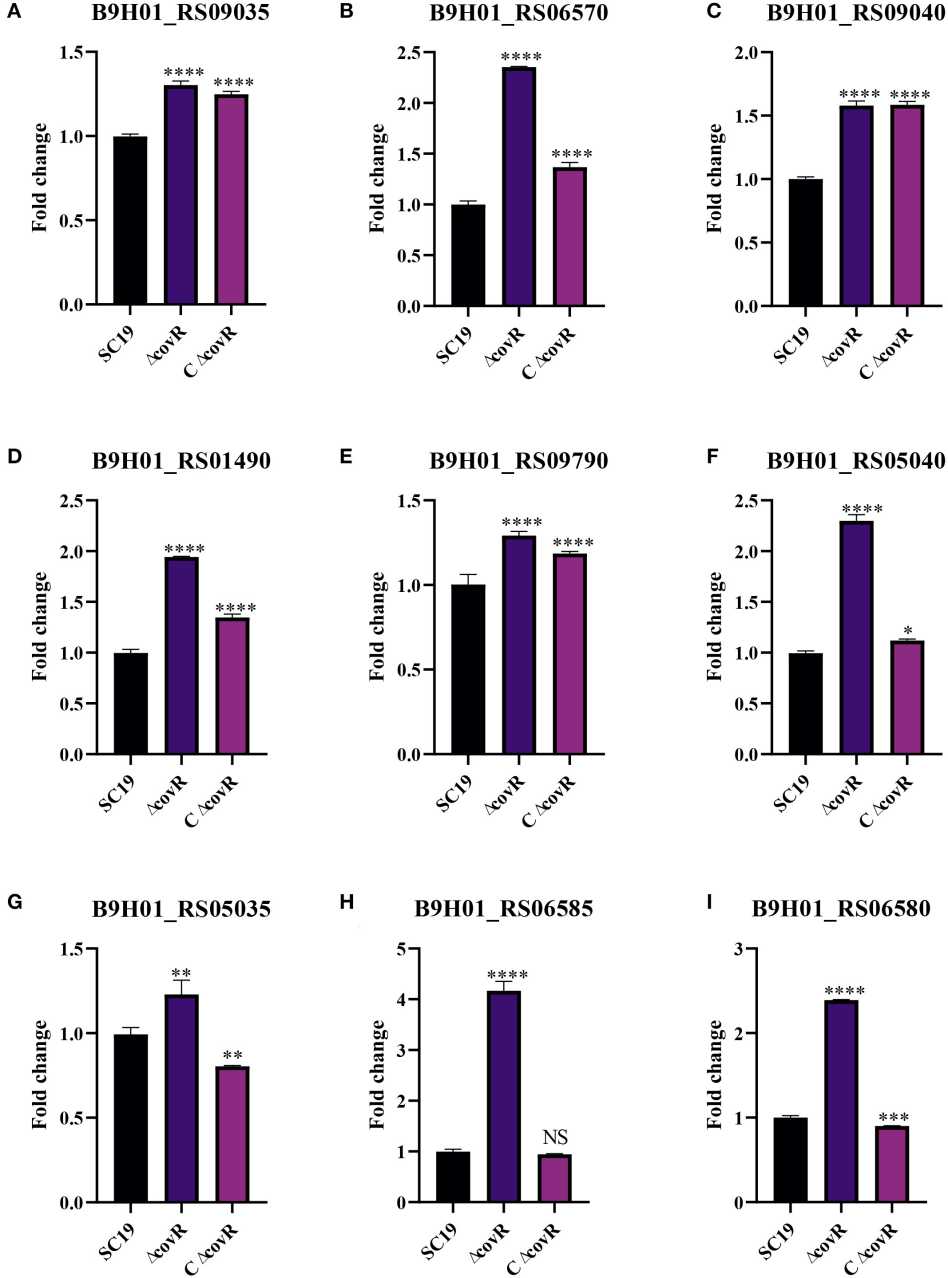
The qRT-PCR validation of genes related to the growth of *S. suis* 2. **(A)** Gene expression levels of B9H01_RS09035. **(B)** Gene expression levels of B9H01_RS06570. **(C)** Gene expression levels of B9H01_RS09040. **(D)** Gene expression levels of B9H01_RS01490. **(E)** Gene expression levels of B9H01_RS09790. **(F)** Gene expression levels of B9H01_RS05040. **(G)** Gene expression levels of B9H01_RS05035. **(H)** Gene expression levels of B9H01_RS06585. **(I)** Gene expression levels of B9H01_RS06580. *p < 0.05, **p < 0.01, ***p < 0.001, ****p < 0.0001.

**Figure 5:**
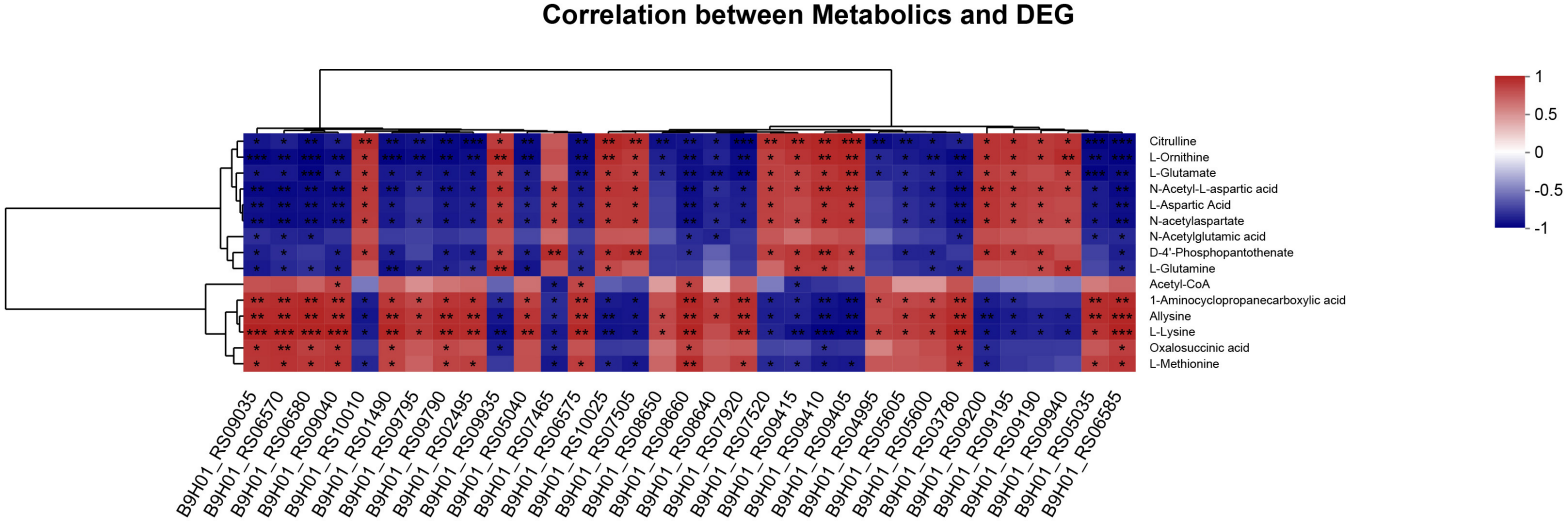
Correlation analysis of DEGs and DAMs in the mutant Δ*covR* and SC19 strains. The levels of correlation of each sample were indicated by the color, from high (red) to low (blue), and the correlation coefficient between samples was represented by the Z-score. *p < 0.05, **p < 0.01, ***p < 0.001.

To validate the accuracy of the identified DEGs by RNA-seq, nine DEGs were validated by qRT-PCR (Figure 4). Consistent with the RNA-Seq results (Supplementary Table S3), qRT-PCR results showed that the expression levels of the identified genes in the mutant Δ*covR* strain were significantly increased compared with those in the SC19 strain. Further research showed that all the gathered data from metabolome and transcriptome profiles had high reliability according to the correlation analysis (Figure 5). We could conclude that variations in metabolite accumulation between SC19 and mutant Δ*covR* strains were closely regulated by different gene expressions.

## Discussion

Prior studies have reported the importance of the TCS CovR in *S. suis* 2 virulence and growth characteristics (14, 22–25). However, the mechanism by which CovR deletion affects the growth of *S. suis* 2 is not yet clear.

### *CovR* is essential for the growth of *S. suis* 2

First, we constructed the *covR* deletion mutant strain. We discovered that the mutant *covR* grew significantly more slowly than the parental strain SC19 on the growth curve (Figure 1A) and it also growth slow on the TSA plate (Figure 1B). This phenomenon was also presented by Pan et al. (22). To further understand the mechanism of poor growth of the Δ*covR* mutant, transcriptome and metabolome profiling were performed.

### The poor growth of Δ*covR* was related to energy metabolism

Energy metabolism, including the tricarboxylic acid cycle (TCA), pentose phosphate pathway, and glycolysis pathway, is one of the most important metabolic pathways in the growth of microorganisms. The TCA is the main pathway by which many substrates, including glycogen, fatty acids, and proteins, enter central carbon metabolism via acetyl-coenzyme A (acetyl-CoA) (26). It is also the main pathway that provides energy for the biosynthesis of complex macromolecules such as glycogen, amino acids, and proteins (27). As we all know, acetyl-CoA and oxalosuccinic acid are the intermediate substances in the TCA, and phosphopantothenate is considered a precursor to the synthesis of coenzyme A (28).

In our study, the metabolism profile showed that oxalosuccinic acid and acetyl-CoA were up-accumulated and D-4'-Phosphopantothenate was down-accumulated. However, the transcriptome profile showed that there were no significant changes in gene expression levels of acetyl-CoA and oxalosuccinic acid between mutant Δ*covR* and SC19 strains. The reason for the up-accumulation of acetyl-CoA and oxalosuccinic acid may be due to less consumption rather than excessive production. The D-4'-Phosphopantothenate was down-accumulated in metabolism and down-expression in the transcriptome, which means there were fewer precursors of coenzyme A, but the level of acetyl-CoA was still up-accumulated. Less consumption is the main reason for the up-accumulation of acetyl-CoA and oxaloacetylsuccinic acid in the mutant Δ*covR* strain. Therefore, the mutant Δ*covR* consumed less acetyl-CoA compared to SC19 and caused less energy supply to the biosynthesis of complex macromolecules, which resulted in poor growth.

### The poor growth of mutant Δ*covR* was related to amino acid biosynthesis and metabolism

Amino acid synthesis and metabolism play a key role in bacterial growth. There are over 300 naturally occurring amino acids, and only 22 constitute the monomer units of proteins, which can be classified as acidic or basic and hydrophilic or hydrophobic according to their respective side chain R (29). 1-aminocyclopropanecarboxylic acid (ACC) is a cyclopropane-containing non-proteinogenic amino acid isolated from various fruits and plant tissues and is considered the immediate precursor of the plant hormone ethylene (30). Recently, ACC has been found to perform similar synthesis machinery to plants in microorganisms (31, 32). Citrulline and ornithine, as we all know, are the intermediate substances of the urea cycle and have recently been reported to be involved in the metabolism of arginine in different bacteria (33). N-acetylglutamate in prokaryotes is also the first intermediate in arginine biosynthesis (34). As we all know, the level of amino acids can influence the amount of aminoacyl-tRNA. Several reports have shown that, in *E. coli* and *S. typhimurium*, the levels of aminoacyl-tRNA synthetases change with the rate of growth in different media (35). Our study found that seven metabolites related to amino acid biosynthesis and metabolism were differently accumulated, including two up-accumulated (1-aminocyclopropanecarboxylic acid and allysine) and five down-accumulated (citrulline, L-ornithine, N-acetylaspartate, N-acetyl-L-aspartic acid, and N-acetylglutamic acid) in mutant Δ*covR* compared to *S. suis* 2 SC19. The down-accumulation of citrulline, ornithine, and N-acetylglutamate were all involved in arginine metabolism, which means that arginine may play a key role in the growth of SC19.

The *argF*, as a gene code ornithine carbamoyltransferase, was downregulated in mutant Δ*covR* compared to SC19 in the transcriptome. Ornithine carbamoyltransferase can catalyze the condensation of ornithine with carbamoyl phosphate to form the amino acid citrulline, which is subsequently delivered to the cytosol (36). In bacteria, urea and ornithine produced by the decomposition of arginine can be further utilized as nitrogen and carbon sources (37). In other words, the mutant Δ*covR* produced less arginine than *S. suis* 2 SC19, which decreased the availability of nitrogen for the biosynthesis of amino acids. Thus, the CovR deletion influenced the rate of amino acid biosynthesis, resulting in poor growth of *S. suis* 2.

### Poor growth of mutant was related to aminoacyl-tRNA biosynthesis

Aminoacyl-tRNA synthetases play a key role in the process of aminoacyl-tRNA biosynthesis, which produces aminoacyl-tRNA, AMP, and PPi by esterifying an amino acid to the 3′ end of a tRNA and hydrolyzing one molecule of ATP (38). Previous studies showed that translation elongation, which is a process of the aminoacyl-tRNA translocation of the ribosome, is an essential step in protein synthesis (39), and translation is the key to defining the growth rate (40). Therefore, the quantity of aminoacyl-tRNA will have an impact on the pace of bacterial growth.

In our study, the aminoacyl-tRNA biosynthesis related to metabolism (L-methionine, L-lysine, L-aspartic Acid, L-glutamate, and L-glutamine) is down-accumulated except L-methionine in the mutant Δ*covR* strain. Meanwhile, the amino acid ABC transporter substrate-binding protein genes (B9H01_RS09940) and the amino acid ABC transporter permease gene (B9H01_RS09935) were downregulated, and the amino acid ABC transporter ATP-binding protein genes (B9H01_RS06570, B9H01_RS09040, and B9H01_RS05040) were upregulated in mutant Δ*covR* compared to SC19. The amino acid ABC transporter is a kind of active transporter for amino acid uptake (41). Therefore, we assume that mutant Δ*covR* has less amino acid ABC transporters that involve in amino acid uptake, thus expressing more ATP-binding proteins to transport more amino acids to maintain homeostasis, but still making the imbalance of aminoacyl tRNA biosynthesis, ultimately leading to slower growth.

## Conclusion

**Figure 6:**
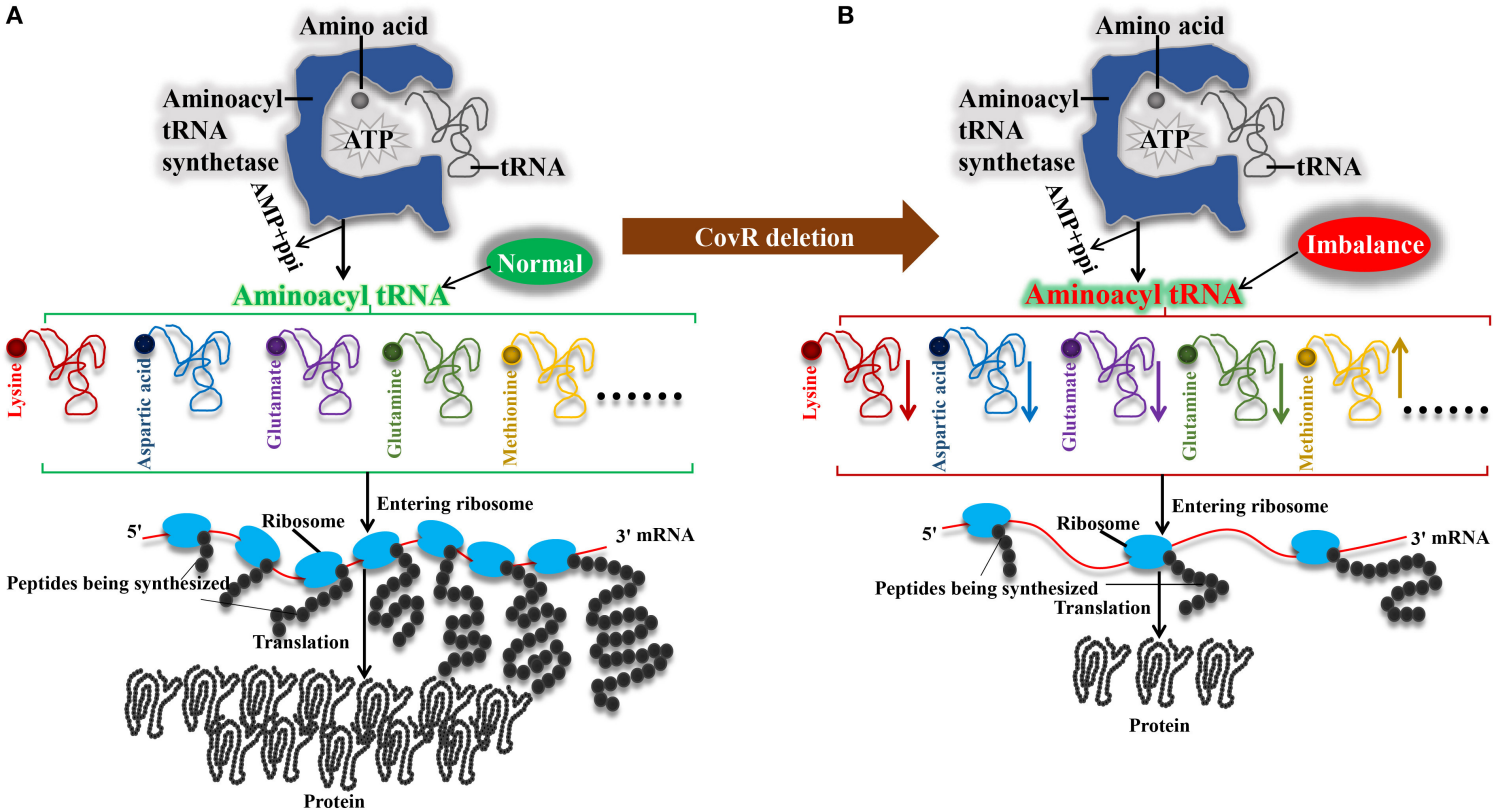
Mechanisms of the poor growth of *Streptococcus suis* serotype 2 after orphan response regulator CovR deletion. **(A)** Synthesis of aminoacyl tRNAs in SC19 in Δ*covR* strain. **(B)** Synthesis of aminoacyl tRNAs in Δ*covR* strain.

The mechanism of slow growth of Δ*covR* was proposed in Figure 6 can be explained as follows: first, the mutant Δ*covR* exhibits reduced consumption of acetyl-CoA, resulting in a decrease in ATP production for biosynthesis. This decrease in ATP production, in turn, leads to a shortage of nitrogen resources due to reduced arginine biosynthesis, which consequently slows down the rate of amino acid biosynthesis. Ultimately, the combination of decreased ATP and amino acid production results in a reduced availability of aminoacyl tRNA, leading to poor growth in mutant Δ*covR*.

## Data availability statement

The original contributions presented in the study are publicly available. This data can be found at: https://www.ebi.ac.uk/metabolights, MTBLS8495 and https://www.ncbi.nlm.nih.gov/, PRJNA879942.

## Author contributions

YZ: Data curation, Funding acquisition, Writing—original draft, Writing—review & editing. BZ: Data curation, Formal analysis, Writing—original draft, Writing—review & editing. YX: Data curation, Formal analysis, Writing—original draft, Methodology, Writing—review & editing. RL: Writing—review & editing, Data curation. HL: Data curation, Formal analysis, Writing—review & editing. PW: Methodology, Writing—review & editing. XY: Writing—review & editing.

## References

[1] Feng Y Zhang H Wu Z Wang S Cao M Hu D. *Streptococcus suis* infection: an emerging/reemerging challenge of bacterial infectious diseases? Virulence. (2014) 5:477–97. 10.4161/viru.28595 24667807 PMC4063810

[2] Zhang S Sella M Sianturi J Priegue P Shen D Seeberger PH. Discovery of oligosaccharide antigens for semi-synthetic glycoconjugate vaccine leads against *Streptococcus suis* serotypes 2, 3, 9 and 14. Angewandte Chemie. (2021) 60:14679–92. 10.1002/anie.202103990 33852172 PMC8252040

[3] Wang Y Wang Y Sun L Grenier D Yi L. *Streptococcus suis* biofilm: regulation, drug-resistance mechanisms, and disinfection strategies. Appl Microbiol Biotechnol. (2018) 102:9121–9. 10.1007/s00253-018-9356-z 30209548

[4] Segura M Calzas C Grenier D Gottschalk M. Initial steps of the pathogenesis of the infection caused by *Streptococcus suis*: fighting against nonspecific defenses. FEBS Lett. (2016) 590:3772–99. 10.1002/1873-3468.12364 27539145

[5] Okura M Osaki M Nomoto R Arai S Osawa R Sekizaki T. Current taxonomical situation of Streptococcus suis. Pathogens. (2016) 5:45. 10.3390/pathogens5030045 27348006 PMC5039425

[6] Goyette-Desjardins G Auger JP Xu J Segura M Gottschalk M. *Streptococcus suis*, an important pig pathogen and emerging zoonotic agent-an update on the worldwide distribution based on serotyping and sequence typing. Emerg Microb Inf. (2014) 3:e45. 10.1038/emi.2014.45 26038745 PMC4078792

[7] He LY Le YJ Guo Z Li S Yang XY. The role and regulatory network of the ciarh two-component system in *Streptococcal species*. Front Microbiol. (2021) 12:693858. 10.3389/fmicb.2021.693858 34335522 PMC8317062

[8] Gao R Stock AM. Biological insights from structures of two-component proteins. Ann Rev Microbiol. (2009) 63:133–54. 10.1146/annurev.micro.091208.073214 19575571 PMC3645274

[9] Gotoh Y Eguchi Y Watanabe T Okamoto S Doi A Utsumi R. Two-component signal transduction as potential drug targets in pathogenic bacteria. Curr Opin Microbiol. (2010) 13:232–9. 10.1016/j.mib.2010.01.008 20138000

[10] Langshaw EL Pandey M Good MF. Cellular interactions of covr/s mutant group A *Streptococci*. Microbes Infection. (2018) 20:531–5. 10.1016/j.micinf.2017.12.009 29287985

[11] Dmitriev A Mohapatra SS Chong P Neely M Biswas S Biswas I. CovR-controlled global regulation of gene expression in *Streptococcus mutans*. PLoS ONE. (2011) 6:e20127. 10.1371/journal.pone.0020127 21655290 PMC3105014

[12] Horstmann N Tran CN Brumlow C DebRoy S Yao H Nogueras Gonzalez G. Phosphatase activity of the control of virulence sensor kinase *covs* is critical for the pathogenesis of group A *Streptococcus*. PLoS Pathog. (2018) 14:e1007354. 10.1371/journal.ppat.1007354 30379939 PMC6231683

[13] Horstmann N Myers KS Tran CN Flores AR Shelburne Iii SA. CovS inactivation reduces covr promoter binding at diverse virulence factor encoding genes in group A *Streptococcus*. PLoS Pathog. (2022) 18:e1010341. 10.1371/journal.ppat.1010341 35180278 PMC8893699

[14] Lamy MC Zouine M Fert J Vergassola M Couve E Pellegrini E. Covs/covr of group B *Streptococcus*: a two-component global regulatory system involved in virulence. Mol Microbiol. (2004) 54:1250–68. 10.1111/j.1365-2958.2004.04365.x 15554966

[15] Ilan O Bloch Y Frankel G Ullrich H Geider K Rosenshine I. Protein tyrosine kinases in bacterial pathogens are associated with virulence and production of exopolysaccharide. EMBO J. (1999) 18:3241–8. 10.1093/emboj/18.12.3241 10369665 PMC1171405

[16] Hu F Zhang Y Liu Q Wang Z. PurA facilitates *Edwardsiella piscicida* to escape NF-κB signaling activation. Fish Shellfish Immunol. (2022) 124:254–60. 10.1016/j.fsi.2022.04.001 35395412

[17] Li X Li Q Zhang Z Wang C Huo X Lai H. Canagliflozin inhibited the activity of hemolysin and reduced the inflammatory response caused by *Streptococcus suis*. Int J Mol Sci. (2023) 24:13074. 10.3390/ijms241713074 37685881 PMC10487456

[18] Rodnina MV. Translation in Prokaryotes. Cold Spring Harb Perspect Biol. (2018) 10:a032664. 10.1101/cshperspect.a032664 29661790 PMC6120702

[19] Li W Yin Y Meng Y Ma Z Lin H Fan H. The phosphorylation of phosphoglucosamine mutase GlmM by Ser/Thr kinase STK mediates cell wall synthesis and virulence in *Streptococcus suis* serotype 2. Vet Microbiol. (2021) 258:109102. 10.1016/j.vetmic.2021.109102 33991786

[20] Zhang YY Li R Li Q Zhu YW Yang XP Zhao D. Orphan response regulator CovR plays positive regulative functions in the survivability and pathogenicity of *Streptococcus suis* serotype 2 isolated from a pig. BMC Vet Res. (2023). 10.1186/s12917-023-03808-9 PMC10664645 37990198

[21] Rio DC Ares M Hannon GJ Nilsen TW. Purification of RNA using TRIzol (TRI reagent). Cold Spring Harb Protoc. (2010) 6:pdb.prot5439. 10.1101/pdb.prot5439 20516177

[22] Pan X Ge J Li M Wu B Wang C Wang J. The orphan response regulator CovR: a globally negative modulator of virulence in *Streptococcus suis* serotype 2. J Bacteriol. (2009) 191:2601–12. 10.1128/JB.01309-08 19181815 PMC2668425

[23] Jiang SM Cieslewicz MJ Kasper DL Wessels MR. Regulation of virulence by a two-component system in group B *Streptococcus*. J Bacteriol. (2005) 187:1105–13. 10.1128/JB.187.3.1105-1113.2005 15659687 PMC545708

[24] Alam FM Turner CE Smith K Wiles S Sriskandan S. Inactivation of the covr/s virulence regulator impairs infection in an improved murine model of *Streptococcus pyogenes* naso-pharyngeal infection. PLoS ONE. (2013) 8:e61655. 10.1371/journal.pone.0061655 23637876 PMC3636223

[25] Lun S Willson PJ. Expression of green fluorescent protein and its application in pathogenesis studies of serotype 2 *Streptococcus suis*. J Microbiol Methods. (2004) 56:401–12. 10.1016/j.mimet.2003.11.012 14967232

[26] Khomyakova M Bükmez Ö Thomas LK Erb TJ Berg IAA. Methylaspartate cycle in haloarchaea. Science. (2011) 331:334–7. 10.1126/science.1196544 21252347

[27] Judge A Dodd MS. Metabolism. Essays Biochem. (2020) 64:607–47. 10.1042/EBC20190041 32830223 PMC7545035

[28] Thomès L Lescure A. Mosaic evolution of the phosphopantothenate biosynthesis pathway in bacteria and archaea. Genome Biol Evol. (2021) 13:262. 10.1093/gbe/evaa262 33320181 PMC7883664

[29] Bullwinkle TJ Ibba M. Translation quality control is critical for bacterial responses to amino acid stress. Proc Nat Acad Sci U S A. (2016) 113:2252–7. 10.1073/pnas.1525206113 26858451 PMC4776511

[30] Wessjohann LA Brandt W Thiemann T. Biosynthesis and metabolism of cyclopropane rings in natural compounds. Chem Rev. (2003) 103:1625–48. 10.1021/cr0100188 12683792

[31] Zha L Jiang Y Henke MT Wilson MR Wang JX Kelleher NL. Colibactin assembly line enzymes use s-adenosylmethionine to build a cyclopropane ring. Nat Chem Biol. (2017) 13:1063–5. 10.1038/nchembio.2448 28805802 PMC5657534

[32] Maruyama C Chinone Y Sato S Kudo F Ohsawa K Kubota J. C-methylation of s-adenosyl-l-methionine occurs prior to cyclopropanation in the biosynthesis of 1-amino-2-methylcyclopropanecarboxylic acid (norcoronamic acid) in a bacterium. Biomolecules. (2020) 10:775. 10.3390/biom10050775 32429436 PMC7277169

[33] Eick S Lussi A. Arginine: a weapon against cariogenic biofilm? Oral Biofilms. (2021) 29:80–90. 10.1159/000510203 33427222

[34] Caldovic L Tuchman M. N-acetylglutamate and its changing role through evolution. Biochem. J. (2003) 372:279–90. 10.1042/BJ20030002 12633501 PMC1223426

[35] Parker J Neidhardt FC. Metabolic regulation of aminoacyl-tRNA synthetase formation in bacteria. Biochem Biophys Res Commun. (1972) 49:495–501. 10.1016/0006-291X(72)90438-X 4565494

[36] Wraith J. Ornithine carbamoyltransferase deficiency. Arch Dis Child. (2001) 84:84–8. 10.1136/adc.84.1.84 11124797 PMC1718609

[37] Hernández VM Arteaga A Dunn MF. Diversity, properties and functions of bacterial arginases. FEMS Microbiol Rev. (2021) 45:304. 10.1093/femsre/fuab034 34160574

[38] Gomez MAR Ibba M. Aminoacyl-trna synthetases. RNA. (2020) 26:910–36. 10.1261/rna.071720.119 32303649 PMC7373986

[39] Xu B Liu L Song G. Functions and regulation of translation elongation factors. Front. Mol. Biosci. (2021) 8:816398. 10.3389/fmolb.2021.816398 35127825 PMC8807479

[40] Belliveau NM Chure G Hueschen CL Garcia HG Kondev J Fisher DS. Fundamental limits on the rate of bacterial growth and their influence on proteomic composition. Cell Syst. (2021) 12:924–44. 10.1016/j.cels.2021.06.002 34214468 PMC8460600

[41] Picossi S Montesinos ML Pernil R. Abc-type neutral amino acid permease n-i is required for optimal diazotrophic growth and is repressed in the heterocysts of *Anabaena sp*. strain pcc 7120. Mol Microbiol. (2005) 57:1582–92. 10.1111/j.1365-2958.2005.04791.x 16135226

