# Supplementary material for: Transcriptome and metabolome profiling to elucidate the mechanism underlying the poor growth of Streptococcus suis serotype 2 after orphan response regulator CovR deletion
Source: Front Vet Sci. 2023 Nov 7;10:1280161. doi: 10.3389/fvets.2023.1280161 (PMC10661955; doi:10.3389/fvets.2023.1280161)
Supplement: Supplementary file 1 [file Data_Sheet_1.docx]

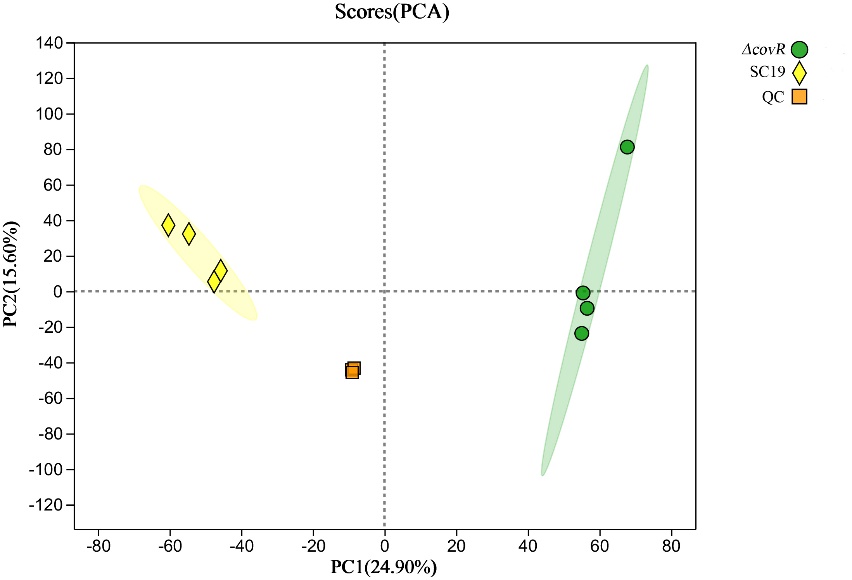


**Supplementary figure S1** Principal component analysis of metabolites identified from Δ *covR* and SC19. Equal volumes of Δ *covR* and SC19 samples were mixed to generate a quality control (QC）
