# Supplementary material for: Transcriptome and metabolome profiling to elucidate the mechanism underlying the poor growth of Streptococcus suis serotype 2 after orphan response regulator CovR deletion
Source: Front Vet Sci. 2023 Nov 7;10:1280161. doi: 10.3389/fvets.2023.1280161 (PMC10661955; doi:10.3389/fvets.2023.1280161)
Supplement: Supplementary file 2 [file Table_1.docx]

**Supplementary table S1** The list of 514 tentatively identified metabolites in the mutants and WT based upon searches against the HMDB and Metlin databases.

| Metabolite | Mode | M/Z | Adducts | Formula | Retention time | RSD |
| --- | --- | --- | --- | --- | --- | --- |
| Asn Pro | pos | 252.0949 | M+Na, M+K, 2M+Na, M+2Na-H, M+H, M+H-H2O | C9H15N3O4 | 1.1632 | 0.001653 |
| Porson | pos | 387.1795 | M+H, M+K, M+NH4, M+Na, M+H-H2O | C22H26O6 | 5.6099 | 0.021601 |
| Hydroxyprolyl-Hydroxyproline | pos | 267.0946 | M+Na, M+K, M+2Na-H, M+H, M+H-2H2O | C10H16N2O5 | 0.7576 | 0.001999 |
| 1-Aminocyclopropanecarboxylic acid | pos | 244.1288 | 2M+H, 2M+ACN+Na, 2M+Na, 2M+ACN+H, M+ACN+H | C4H7NO2 | 0.722 | 0.00711 |
| 4-hydroxy Nonenal Alkyne | pos | 194.1149 | M+H, M+ACN+H, M+NH4, M+H-H2O, M+H-2H2O | C9H12O2 | 7.9874 | 0.003054 |
| Betaine | pos | 118.0863 | M+Na, 2M+Na, 2M+H, M+H | C5H11NO2 | 0.648 | 0.001114 |
| Citrulline | pos | 198.0847 | M+Na, M+2Na-H, M+K, M+H | C6H13N3O3 | 0.666 | 0.001118 |
| Nicotinamide adenine dinucleotide (NAD) | pos | 664.1156 | M+H, M+2Na-H, M+Na, M+2H | C21H27N7O14P2 | 0.722 | 0.017711 |
| (E)-3,7-Dimethyl-2,6-octadienyl 3-methylbutanoate | pos | 256.2266 | M+H, M+NH4, M+H-H2O, M+H-2H2O | C15H26O2 | 6.4289 | 0.005378 |
| 3,7-Dimethyl-2E,6-octadienyl acetate | pos | 214.1798 | M+H-H2O, M+NH4, M+H, M+H-2H2O | C12H20O2 | 4.9465 | 0.003352 |
| Linoleamide | pos | 280.2629 | M+H, M+Na, 2M+H, M+H-H2O | C18H33NO | 8.3695 | 0.011952 |
| 3-ketosphingosine | pos | 298.2734 | M+Na, M+ACN+H, M+K, M+H | C18H35NO2 | 7.6618 | 0.00244 |
| 8(R)-Hydroperoxylinoleic acid | pos | 313.2367 | M+H, M+Na, M+NH4, M+H-H2O | C18H32O4 | 7.4233 | 0.001574 |
| Bis(2-ethylhexyl) phthalate | pos | 391.2835 | M+Na, M+K, 2M+Na, M+H | C24H38O4 | 9.211 | 0.007496 |
| Propyl 2,4-decadienoate | pos | 228.1954 | M+H-H2O, M+NH4, M+H, M+H-2H2O | C13H22O2 | 5.4854 | 0.00234 |
| Auberganol | pos | 258.2424 | M+H-H2O, M+H, M+NH4, M+H-2H2O | C15H28O2 | 6.1365 | 0.004466 |
| 4,5-Dihydrovomifoliol | pos | 244.1903 | M+H-H2O, M+NH4, M+H, M+H-2H2O | C13H22O3 | 5.1445 | 0.010892 |
| 12-amino-dodecanoic acid | pos | 216.1955 | M+H, M+Na, M+ACN+H, M+H-H2O | C12H25NO2 | 4.7488 | 0.008877 |
| Glycerophosphocholine | pos | 258.1096 | M+H, M+K, M+Na | C8H20NO6P | 0.6108 | 0.02983 |
| Lactulose | pos | 325.1113 | M+Na, M+K, M+H-H2O | C12H22O11 | 0.6839 | 0.007242 |
| 3-Deoxy-D-glycero-D-galacto-2-nonulosonic acid | pos | 291.0694 | M+Na, M+K, M+H | C9H16O9 | 0.8122 | 0.010448 |
| Glu Gly Asn | pos | 319.1242 | M+H, M+Na, M+K | C11H18N4O7 | 0.7038 | 0.008559 |
| 7,9-decadienoic acid | pos | 186.1486 | M+H, M+NH4, M+H-H2O | C10H16O2 | 3.3138 | 0.006608 |
| 6-Decynoic acid | pos | 186.1486 | M+H, M+NH4, M+H-H2O | C10H16O2 | 3.6918 | 0.002802 |
| (3R,5Z)-5-Octene-1,3-diol | pos | 109.1013 | M+H-2H2O, 2M+K, M+H-H2O | C8H16O2 | 2.9609 | 0.022045 |
| 2,4-Hexadienyl butyrate | pos | 133.1011 | M+H-2H2O, M+H-H2O, M+H | C10H16O2 | 5.4854 | 0.005959 |
| 2-Hydroxyacorenone | pos | 254.2109 | M+H, M+NH4, M+H-H2O | C15H24O2 | 5.9431 | 0.010021 |
| 5,9:6,9-Diepoxy-3-megastigmene | pos | 209.1534 | M+H-H2O, M+CH3OH+H, M+H | C13H20O2 | 6.0932 | 0.008785 |
| C9:2n-2,4 | pos | 155.1064 | M+H, M+NH4, M+H-H2O | C9H14O2 | 7.9874 | 0.007726 |
| 1-heneicosanoyl-glycero-3-phosphate | pos | 522.3546 | M+ACN+H, M+ACN+Na, M+H-2H2O | C24H49O7P | 8.1148 | 0.019248 |
| Methyl-delta-ionone | pos | 224.2005 | M+H-H2O, M+H, M+NH4 | C14H22O | 8.3695 | 0.010147 |
| Beta-Ionol | pos | 212.2005 | M+H, M+NH4, M+H-H2O | C13H22O | 8.7086 | 0.006868 |
| 9,12,15-Octadecatrien-1-ol | pos | 282.2786 | M+H, M+NH4, M+H-H2O | C18H32O | 8.7721 | 0.002413 |
| Geranylcitronellol | pos | 310.3097 | M+H, M+NH4, M+H-H2O | C20H36O | 9.17 | 0.005727 |
| 3-Methylglutaric acid | pos | 129.0546 | M+H-H2O, M+H, M+H-2H2O | C6H10O4 | 9.3157 | 0.005268 |
| (+/-)-1,4-Nonanediol diacetate | pos | 245.1742 | M+H-H2O, M+H, 2M+K | C13H24O4 | 9.7991 | 0.010998 |
| DG(18:0/18:3(6Z,9Z,12Z)/0:0) | pos | 619.5289 | M+H, M+Na, M+NH4 | C39H70O5 | 10.1615 | 0.018339 |
| DG(18:0/18:2(9Z,12Z)/0:0) | pos | 638.5708 | M+NH4, M+Na, M+H | C39H72O5 | 10.3539 | 0.006285 |
| Sebacic acid | pos | 185.117 | M+H-H2O, M+H, M+H-2H2O | C10H18O4 | 9.7584 | 0.008512 |
| 2-Hydroxy-2,6,6-trimethylcyclohexanone | pos | 139.1115 | M+H-H2O, M+H, M+H-2H2O | C9H16O2 | 9.7584 | 0.011411 |
| (4E,8E,10E-d18:3)sphingosine | pos | 296.2579 | M+H, M+K, M+Na | C18H33NO2 | 7.3387 | 0.001028 |
| 6-[5]-ladderane-1-hexanol | pos | 278.2474 | M+H, M+NH4, M+H-H2O | C18H28O | 7.3173 | 0.0032 |
| Mevaldate | pos | 147.065 | M+H-H2O, M+H, M+H-2H2O | C6H10O4 | 5.6099 | 0.018112 |
| 5E,7Z-Dodecadienal | pos | 198.185 | M+H-H2O, M+H, M+NH4 | C12H20O | 5.1445 | 0.007474 |
| 3,6,7-Trihydroxy-4'-methoxyflavone 7-rhamnoside | pos | 429.1173 | M+H-H2O, M+H, M+Na | C22H22O10 | 4.5943 | 0.008495 |
| (S)-9-Hydroxy-10-undecenoic acid | pos | 183.1377 | M+H-H2O, M+H, M+H-2H2O | C11H20O3 | 4.3537 | 0.003492 |
| Gln Pro | pos | 226.1182 | M+H-H2O, M+Na, M+H | C10H17N3O4 | 1.2624 | 0.003098 |
| Threoninyl-Glutamate | pos | 213.0868 | M+H, M+K, M+H-2H2O | C9H16N2O6 | 0.722 | 0.00468 |
| Inosine | pos | 269.0875 | M+H, M+Na, 2M+H | C10H12N4O5 | 1.6733 | 0.012016 |
| Asp Met | pos | 265.0848 | M+H, M+Na, M+H-H2O | C9H16N2O5S | 1.7542 | 0.00682 |
| Cinnamic acid | pos | 166.0861 | M+H-H2O, M+H, M+NH4 | C9H8O2 | 1.8165 | 0.020861 |
| Pro Leu | pos | 229.1543 | M+Na, 2M+H, M+H | C11H20N2O3 | 1.895 | 0.010859 |
| 3-amino-2-naphthoic acid | pos | 188.0704 | M+H, M+NH4, M+H-H2O | C11H9NO2 | 2.0642 | 0.002351 |
| Asp Glu Leu | pos | 376.1707 | M+H, M+Na, M+H-H2O | C15H25N3O8 | 2.1452 | 0.005749 |
| (+/-)-2-Hydroxy-4-(methylthio)butanoic acid | pos | 192.0687 | M+ACN+H, M+ACN+Na, M+H-H2O | C5H10O3S | 2.187 | 0.006171 |
| (E)-hex-2-enedioic acid | pos | 127.039 | M+H-H2O, M+H, M+H-2H2O | C6H8O4 | 0.4248 | 0.022626 |
| (3b,20R,22R)-3,20,27-Trihydroxy-1-oxowitha-5,24-dienolide 3-glucoside | pos | 635.3389 | M+H, M+Na, M+K | C34H50O11 | 2.6279 | 0.022153 |
| Leu Pro Thr | pos | 330.2016 | M+H, M+K, M+Na | C15H27N3O5 | 2.6725 | 0.008839 |
| L-Ornithine | pos | 133.0971 | M+H, M+K | C5H12N2O2 | 0.6293 | 0.003806 |
| 4-Methylene-L-glutamine | pos | 159.0762 | M+H, M+Na | C6H10N2O3 | 0.666 | 0.003102 |
| 2-Aminobut-2-enoate | pos | 102.0552 | M+H-H2O, M+H | C4H7NO2 | 0.6839 | 0.001398 |
| Glu Ser | pos | 235.092 | M+H, M+Na | C8H14N2O6 | 0.7038 | 0.002594 |
| Pro Pro | pos | 213.123 | M+H, M+Na | C10H16N2O3 | 0.7754 | 0.005361 |
| D-Pipecolic acid | pos | 130.0862 | M+H, M+CH3OH+H | C6H11NO2 | 0.7398 | 0.007893 |
| Beta-Nicotinamide mononucleotide | pos | 335.0632 | M+H, M+Na | C11H15N2O8P | 0.722 | 0.024465 |
| P-Tolualdehyde | pos | 121.0648 | M+H-H2O, M+H | C8H8O | 5.7543 | 0.019708 |
| 2-ISOPROPYL-3-METHOXYCINNAMIC ACID | pos | 221.1169 | M+H-H2O, M+H | C13H16O3 | 6.7973 | 0.006302 |
| 2-Methyl-3-(2-pentenyl)-2-cyclopenten-1-one | pos | 182.1537 | M+H-H2O, M+NH4 | C11H16O | 7.5525 | 0.006261 |
| Decenedioic acid | pos | 201.1119 | M+H-H2O, M+H | C10H16O4 | 7.4233 | 0.004667 |
| Serratol | pos | 308.2941 | M+H, M+NH4 | C20H34O | 7.7269 | 0.004688 |
| PC(18:2/0:0) | pos | 520.3391 | M+H, M+Na | C26H50NO7P | 7.7704 | 0.006087 |
| Linoleoyl Ethanolamide | pos | 324.289 | M+H, M+Na | C20H37NO2 | 7.7485 | 0.009224 |
| PC(16:0/0:0) | pos | 496.339 | M+H, M+Na | C24H50NO7P | 7.9874 | 0.007149 |
| Cis-3-Hexenyl pyruvate | pos | 188.1279 | M+H, M+NH4 | C9H14O3 | 8.0083 | 0.006707 |
| Dihomo-gamma-Linolenoyl ethanolamide | pos | 350.3047 | M+H, M+Na | C22H39NO2 | 8.0509 | 0.002715 |
| 3-(5,6,6-Trimethylbicyclo[2.2.1]hept-1-yl)cyclohexanol | pos | 254.2473 | M+H-H2O, M+NH4 | C16H28O | 8.2199 | 0.024739 |
| Docosatrienoic acid | pos | 352.3203 | M+H, M+NH4 | C22H38O2 | 8.263 | 0.006166 |
| Farnesyl acetone | pos | 263.2365 | M+H, M+H-H2O | C18H30O | 8.3695 | 0.014487 |
| Palmitic amide | pos | 256.263 | M+H, 2M+H | C16H33NO | 8.6011 | 0.010656 |
| Oleamide | pos | 563.5501 | M+Na, 2M+H | C18H35NO | 8.794 | 0.008774 |
| 5-(3E-Pentenyl)tetrahydro-2-oxo-3-furancarboxylic acid | pos | 199.0962 | M+H-H2O, M+H | C10H14O4 | 9.1298 | 0.007857 |
| Phthalic acid Mono-2-ethylhexyl Ester | pos | 279.1585 | M+H-H2O, M+H | C16H22O4 | 9.211 | 0.015938 |
| 6-Methoxymellein | pos | 209.0805 | M+H-H2O, M+H | C11H12O4 | 9.2323 | 0.035213 |
| MG(0:0/14:1(9Z)/0:0) | pos | 301.2367 | M+H-H2O, M+H | C17H32O4 | 9.6746 | 0.007616 |
| 9,10-DHOME | pos | 315.2524 | M+H-H2O, M+H | C18H34O4 | 9.7794 | 0.004141 |
| MG(0:0/22:1(13Z)/0:0) | pos | 413.3618 | M+H, M+Na | C25H48O4 | 9.6746 | 0.033248 |
| (2R,3R,4R)-2-Amino-4-hydroxy-3-methylpentanoic acid | pos | 130.0862 | M+H-H2O, M+Na | C6H13NO3 | 14.0184 | 0.011408 |
| 1,2,3-Trihydroxybenzene | pos | 127.039 | M+H-H2O, M+H | C6H6O3 | 15.9391 | 0.013344 |
| 2-(1-Aziridinyl)ethanol | pos | 88.07619 | M+H, M+ACN+H | C4H9NO | 14.0385 | 0.018322 |
| 2-Hydroxypropyl 2-isopropyl-5-methylcyclohexyl carbonate | pos | 259.1899 | M+H-H2O, M+H | C14H26O4 | 9.3157 | 0.008371 |
| 4-Formylsalicylic acid | pos | 149.0231 | M+H-H2O, M+H | C8H6O4 | 9.2323 | 0.005006 |
| 1,4-dimethylcyclohexane | pos | 113.1326 | M+H, M+ACN+H | C8H16 | 9.2323 | 0.006302 |
| Cis-Quinceoxepane | pos | 198.185 | M+H-H2O, M+NH4 | C12H20O | 8.794 | 0.003938 |
| 2-Hexylidenecyclopentanone | pos | 184.1694 | M+H-H2O, M+NH4 | C11H18O | 8.794 | 0.00833 |
| (R)-Carvotanacetone | pos | 170.1537 | M+H-H2O, M+NH4 | C10H16O | 8.794 | 0.007155 |
| 4E,6E,10Z-Hexadecatrien-1-ol | pos | 237.2208 | M+H, M+Na | C16H28O | 8.2199 | 0.011297 |
| DL-2-Aminooctanoic acid | pos | 142.1225 | M+H-H2O, M+H | C8H17NO2 | 7.9874 | 0.021344 |
| Palmitoleoyl Ethanolamide | pos | 320.2555 | M+Na, M+K | C18H35NO2 | 7.1888 | 0.015042 |
| 3-Methyl-alpha-ionyl acetate | pos | 268.2267 | M+H, M+NH4 | C16H26O2 | 6.5149 | 0.00913 |
| 2,5-Dimethylbenzaldehyde | pos | 135.0803 | M+H-H2O, M+H | C9H10O | 6.1785 | 0.007528 |
| 8-Deoxy-11,13-dihydroxygrosheimin | pos | 281.1378 | M+H-H2O, M+H | C15H20O5 | 6.1785 | 0.003885 |
| Asp Gln Glu | pos | 391.1452 | M+H, M+Na | C14H22N4O9 | 1.1038 | 0.021686 |
| Ergothioneine | pos | 230.0954 | M+H, M+K | C9H15N3O2S | 0.722 | 0.025622 |
| 5'-CMP | pos | 324.0585 | M+H, 2M+H | C9H14N3O8P | 1.1248 | 0.001119 |
| L-Aspartic Acid | pos | 134.0447 | M+H-2H2O, M+H | C4H7NO4 | 1.2034 | 0.000571 |
| N-acetylaspartate | pos | 158.0446 | M+H-H2O, M+H | C6H9NO5 | 1.2228 | 0.004588 |
| Adenosine 3'-monophosphate | pos | 348.0696 | M+H, M+Na | C10H14N5O7P | 1.2228 | 0.011028 |
| THTC | pos | 150.0581 | M+H, M+NH4 | C5H8O2S | 1.2228 | 0.003413 |
| Aspartyl-Proline | pos | 231.097 | M+H, M+Na | C9H14N2O5 | 1.2833 | 0.001419 |
| Guanidylic acid (guanosine monophosphate) | pos | 364.0645 | M+H, M+Na | C10H14N5O8P | 1.2624 | 0.00465 |
| 4-Hydroxyproline galactoside | pos | 276.1072 | M+H-H2O, M+K | C11H19NO8 | 1.6173 | 0.009 |
| Thr Pro | pos | 217.1179 | M+H-H2O, M+H | C9H16N2O4 | 1.6363 | 0.002308 |
| Glu Gly Pro | pos | 302.1341 | M+H, M+CH3OH+H | C12H19N3O6 | 1.7542 | 0.011888 |
| Indole-3-acetamide | pos | 175.0864 | M+H-H2O, M+H | C10H10N2O | 1.7747 | 0.011292 |
| (S)-Isosclerone | pos | 196.0965 | M+H, M+NH4 | C10H10O3 | 1.8165 | 0.011767 |
| Normetanephrine | pos | 148.0755 | M+H-2H2O, M+Na | C9H13NO3 | 1.8737 | 0.013782 |
| Tetrahydropentoxyline | pos | 367.1495 | M+H-H2O, M+H | C17H22N2O7 | 1.9809 | 0.021199 |
| Gly Pro Val | pos | 272.1601 | M+H-H2O, M+H | C12H21N3O4 | 1.7963 | 0.013493 |
| Asp Ser Leu | pos | 375.1866 | M+H, M+ACN+H | C13H23N3O7 | 2.0432 | 0.005169 |
| 4-Chlorobenzaldehyde | pos | 141.01 | M+H, M+ACN+H | C7H5ClO | 2.1018 | 0.013654 |
| 1,2-Dihydronaphthalene-1,2-diol | pos | 180.1016 | M+H, M+NH4 | C10H10O2 | 2.1018 | 0.008008 |
| 7-Methylguanosine | pos | 316.1496 | M+H-H2O, M+NH4 | C11H16N5O5+ | 2.208 | 0.005901 |
| Hydroxyamobarbital | pos | 243.1335 | M+H-H2O, M+H | C11H18N2O4 | 2.3848 | 0.009103 |
| L-Asparagine | pos | 133.0607 | M+H, M+K | C4H8N2O3 | 0.6108 | 0.012226 |
| 2-Amino-5-phenylpyridine | pos | 212.118 | M+H, M+ACN+H | C11H10N2 | 2.5166 | 0.000775 |
| Pro Val Pro Val | pos | 411.2594 | M+H, M+Na | C20H34N4O5 | 2.5828 | 0.003448 |
| Pro Val Val | pos | 314.2067 | M+H, M+Na | C15H27N3O4 | 2.7398 | 0.013738 |
| Pro Ser Pro Pro | pos | 397.2074 | M+H-H2O, M+H | C18H28N4O6 | 2.6279 | 0.010146 |
| 4,7-dioxo-octanoic acid | pos | 173.0806 | M+H-H2O, M+H | C8H12O4 | 2.9609 | 0.073673 |
| Methyl 1-methoxy-1H-indole-3-carboxylate | pos | 206.0809 | M+H, M+NH4 | C11H11NO3 | 3.0694 | 0.014881 |
| N-Acetyl-DL-tryptophan | pos | 247.1072 | M+H, M+Na | C13H14N2O3 | 3.3138 | 0.004459 |
| Lumichrome | pos | 243.0872 | M+H, M+Na | C12H10N4O2 | 3.5589 | 0.017944 |
| 5-Hydroxy-p-mentha-6,8-dien-2-one | pos | 333.2011 | M+NH4, 2M+H | C10H14O2 | 3.6254 | 0.010201 |
| 12-hydroxy-10-dodecenoic acid | pos | 215.1638 | M+H-H2O, M+H | C12H22O3 | 4.4628 | 0.004766 |
| 2,2,6,7-Tetramethylbicyclo[4.3.0]nona-1(9),4-dien-8-one | pos | 208.1694 | M+NH4, M+H-H2O | C13H18O | 5.1445 | 0.006487 |
| 2,5-Heptadien-1-ol | pos | 95.08588 | M+H-H2O, M+H | C7H12O | 5.4854 | 0.013278 |
| 2,6-nonadienoic acid | pos | 155.1064 | M+H-H2O, M+H | C9H14O2 | 5.4854 | 0.010976 |
| 3-Methyl-2E-hexenoic acid | pos | 129.091 | M+H-H2O, M+H | C7H12O2 | 5.4854 | 0.009099 |
| 5-Ethyl-2,3-dimethylpyrazine | pos | 154.1337 | M+NH4 | C8H12N2 | 0.6108 | 0.098371 |
| Pro His | pos | 253.129 | M+H | C11H16N4O3 | 0.9366 | 0.001079 |
| Ser Pro | pos | 203.1023 | M+H | C8H14N2O4 | 1.1436 | 0.003632 |
| NIPECOTIC ACID | pos | 130.0862 | M+H | C6H11NO2 | 1.2425 | 0.026998 |
| Allobarbital | pos | 209.0908 | M+H | C10H12N2O3 | 1.2624 | 0.006898 |
| Pyridoxal Phosphate | pos | 248.0313 | M+H | C8H10NO6P | 1.3239 | 0.001963 |
| Ala Pro | pos | 187.1075 | M+H | C8H14N2O3 | 1.3454 | 0.001476 |
| 7-Aminomethyl-7-carbaguanine | pos | 180.0877 | M+H | C7H9N5O | 1.5968 | 0.011296 |
| ADP | pos | 428.036 | M+H | C10H15N5O10P2 | 1.6173 | 0.007356 |
| Lysyl-Proline | pos | 226.1544 | M+H-H2O | C11H21N3O3 | 1.6543 | 0.011466 |
| Thr Ser Thr Pro | pos | 405.1971 | M+H | C16H28N4O8 | 1.6543 | 0.008314 |
| Piperidine | pos | 86.09685 | M+H | C5H11N | 1.6929 | 0.007647 |
| Thymine | pos | 127.0501 | M+H | C5H6N2O2 | 1.7334 | 0.001376 |
| Histidinyl-Isoleucine | pos | 251.1498 | M+H-H2O | C12H20N4O3 | 1.7334 | 0.005196 |
| Asp Met Glu | pos | 394.1271 | M+H | C14H23N3O8S | 1.7542 | 0.00466 |
| 4-Hydroxy-5-phenyltetrahydro-1,3-oxazin-2-one | pos | 158.0599 | M+H-2H2O | C10H11NO3 | 1.7747 | 0.018971 |
| Val Pro Pro | pos | 312.1911 | M+H | C15H25N3O4 | 1.8165 | 0.009781 |
| 3'-Hydroxyhexobarbital | pos | 505.2286 | 2M+H | C12H16N2O4 | 1.8165 | 0.022704 |
| Pro Ile Pro | pos | 326.2067 | M+H | C16H27N3O4 | 1.8343 | 0.016955 |
| Ser Glu Ala Val | pos | 405.1972 | M+H | C16H28N4O8 | 1.8533 | 0.092956 |
| N6,N6-Dimethyladenosine | pos | 296.1348 | M+H | C12H17N5O4 | 1.895 | 0.005282 |
| Ser Leu | pos | 219.1336 | M+H | C9H18N2O4 | 1.9809 | 0.005932 |
| Gly Pro Leu | pos | 286.1755 | M+H | C13H23N3O4 | 2.0229 | 0.010199 |
| 4-formyl Indole | pos | 146.0599 | M+H | C9H7NO | 2.0642 | 0.007612 |
| L-beta-aspartyl-L-leucine | pos | 247.1283 | M+H | C10H18N2O5 | 2.0642 | 0.007461 |
| Leucyl-Gamma-glutamate | pos | 242.1493 | M+H-H2O | C11H21N3O4 | 2.0821 | 0.026147 |
| Pseudouridine | pos | 245.0764 | M+H | C9H12N2O6 | 2.1235 | 0.028557 |
| Asp Gly Ile | pos | 304.1496 | M+H | C12H21N3O6 | 2.1235 | 0.007351 |
| Gly Asp Glu Leu | pos | 433.1921 | M+H | C17H28N4O9 | 2.1235 | 0.004308 |
| 1-(Ethylthio)ethyl methyl disulfide | pos | 201.0446 | M+CH3OH+H | C5H12S3 | 2.1452 | 0.165169 |
| L-phenylalanyl-L-proline | pos | 263.1385 | M+H | C14H18N2O3 | 2.1452 | 0.0091 |
| (+/-)-2-Methylthiazolidine | pos | 104.0531 | M+H | C4H9NS | 2.187 | 0.02024 |
| Asp Pro Ile | pos | 344.1809 | M+H | C15H25N3O6 | 2.2296 | 0.004412 |
| Gamma-Glu-Leu | pos | 261.1441 | M+H | C11H20N2O5 | 2.2517 | 0.013184 |
| N-Deschlorobenzoyl indomethacin | pos | 202.0859 | M+H-H2O | C12H13NO3 | 2.5609 | 0.009756 |
| Hydroxypentobarbital | pos | 243.1335 | M+H | C11H18N2O4 | 2.8071 | 0.016414 |
| 3-Aminoquinoline | pos | 145.0758 | M+H | C9H8N2 | 3.0259 | 0.008713 |
| 1-ACETYLPIPERIDINE | pos | 128.1069 | M+H | C7H13NO | 3.0917 | 0.015593 |
| Isopentenyl adenosine | pos | 336.1659 | M+H | C15H21N5O4 | 3.4699 | 0.011095 |
| 2-(3-Hydroxy-4-methylphenyl)-5-methyl-4-hexen-3-one | pos | 219.1376 | M+H | C14H18O2 | 3.5806 | 0.004803 |
| Triethyl phosphate | pos | 183.0779 | M+H | C6H15O4P | 3.7584 | 0.005129 |
| Ethyl 3-hydroxydodecanoate | pos | 262.2371 | M+NH4 | C14H28O3 | 3.8249 | 0.004727 |
| UMBELLIFERONE | pos | 163.0387 | M+H | C9H6O3 | 4.2433 | 0.005117 |
| 12(S)-HETrE | pos | 345.2374 | M+Na | C20H34O3 | 4.2433 | 0.002053 |
| Dihydrocapsaicin | pos | 325.2479 | M+NH4 | C18H29NO3 | 4.5287 | 0.005648 |
| 2,2,6,7-Tetramethylbicyclo[4.3.0]nona-1(9),4-diene-7,8-diol | pos | 226.1798 | M+NH4 | C13H20O2 | 5.1445 | 0.002254 |
| (9E)-10-nitrooctadecenoic Acid | pos | 310.2347 | M+H-H2O | C18H33NO4 | 5.5063 | 0.007145 |
| Annuionone C | pos | 225.1482 | M+H | C13H20O3 | 5.6713 | 0.012642 |
| Melleolide | pos | 418.2216 | M+NH4 | C23H28O6 | 5.7543 | 0.007739 |
| C17 Sphinganine | pos | 288.2891 | M+H | C17H37NO2 | 6.1996 | 0.010455 |
| Lucidenolactone | pos | 474.2838 | M+NH4 | C27H36O6 | 6.3239 | 0.02022 |
| (-)-Carvomenthone | pos | 172.1693 | M+NH4 | C10H18O | 6.3453 | 0.014998 |
| Cyclododecanone | pos | 200.2006 | M+NH4 | C12H22O | 7.1468 | 0.026568 |
| Pinolenic Acid | pos | 279.2314 | M+H | C18H30O2 | 7.2326 | 0.007724 |
| N,N-dimethyl-Safingol | pos | 330.3358 | M+H | C20H43NO2 | 7.2326 | 0.016636 |
| (E,E)-2,4-Decadienoic isobutylamide | pos | 224.2005 | M+H | C14H25NO | 7.2531 | 0.009262 |
| Eicosanoyl-EA | pos | 356.3517 | M+H | C22H45NO2 | 7.4662 | 0.00611 |
| 2-Acetyl-3,5,5,6,8,8-hexamethyl-5,6,7,8-tetrahydronaphthalene | pos | 276.2317 | M+NH4 | C18H26O | 7.5963 | 0.001424 |
| Nepetaside | pos | 329.1588 | M+H-H2O | C16H26O8 | 7.6399 | 0.003565 |
| Lys Lys Val Lys | pos | 502.373 | M+H | C23H47N7O5 | 7.8364 | 0.000808 |
| Hericene B | pos | 600.4673 | M+NH4 | C37H58O5 | 7.9014 | 0.00396 |
| Ethylbenzene | pos | 107.0857 | M+H | C8H10 | 7.9874 | 0.019806 |
| Tetracosahexaenoic acid | pos | 374.3021 | M+NH4 | C24H36O2 | 8.0296 | 0.009138 |
| Ethyl sorbate | pos | 141.0908 | M+H | C8H12O2 | 8.4534 | 0.007378 |
| Panaxydol linoleate | pos | 540.4462 | M+NH4 | C35H54O3 | 8.6011 | 0.008772 |
| Docosadienoate (22:2n6) | pos | 354.3359 | M+NH4 | C22H40O2 | 8.6232 | 0.008196 |
| (4E)-1-(3,4-dihydroxy-5-methoxyphenyl)dec-4-en-3-one | pos | 325.2004 | M+CH3OH+H | C17H24O4 | 8.7086 | 0.007495 |
| (E)-6,10-Dimethyl-9-methylene-5-undecen-2-one | pos | 226.2161 | M+NH4 | C14H24O | 8.7721 | 0.00917 |
| 3,5,5-Trimethyl-3-cyclohexen-1-one | pos | 156.1381 | M+NH4 | C9H14O | 9.0462 | 0.00138 |
| 4alpha-Carboxy-5alpha-cholesta-8-en-3beta-ol | pos | 463.3769 | M+CH3OH+H | C28H46O3 | 9.379 | 0.008136 |
| 2-Methyl-2-cyclopenten-1-one | pos | 138.0911 | M+ACN+H | C6H8O | 9.4631 | 0.001929 |
| MG(0:0/22:4(7Z,10Z,13Z,16Z)/0:0) | pos | 439.341 | M+CH3OH+H | C25H42O4 | 9.6532 | 0.006535 |
| Methylgingerol | pos | 309.2054 | M+H | C18H28O4 | 9.6959 | 0.0242 |
| 7-Oxo-11-dodecenoic acid | pos | 213.1482 | M+H | C12H20O3 | 9.9677 | 0.009926 |
| Isobutylpropylamine | pos | 116.1434 | M+H | C7H17N | 13.9772 | 0.007803 |
| Pyrrolidine | pos | 72.08144 | M+H | C4H9N | 14.1194 | 0.034564 |
| 3-Pyridinaldehyde | pos | 108.0446 | M+H | C6H5NO | 15.669 | 0.007534 |
| Theobromine | pos | 181.0718 | M+H | C7H8N4O2 | 15.9164 | 0.007806 |
| Caffeine | pos | 195.0875 | M+H | C8H10N4O2 | 15.669 | 0.028486 |
| 5-Methyl-2-furaldehyde | pos | 111.0443 | M+H | C6H6O2 | 14.0969 | 0.002609 |
| 7a,12a-Dihydroxy-cholestene-3-one | pos | 449.3595 | M+CH3OH+H | C27H44O3 | 14.0184 | 0.019347 |
| Fagomine | pos | 148.0966 | M+H | C6H13NO3 | 14.0184 | 0.004031 |
| Cyclohexane | pos | 102.128 | M+NH4 | C6H12 | 13.9772 | 0.002843 |
| Tripropylamine | pos | 144.1745 | M+H | C9H21N | 13.9356 | 0.006194 |
| 2,3-Dimethyl-2-cyclohexen-1-one | pos | 142.1225 | M+NH4 | C8H12O | 13.9356 | 0.005061 |
| Isopropyl linoleate | pos | 323.2938 | M+H | C21H38O2 | 10.1615 | 0.010551 |
| MG(0:0/18:1(11Z)/0:0) | pos | 357.2992 | M+H | C21H40O4 | 9.7584 | 0.005358 |
| 6-Acetyl-1,2,3,4-tetrahydropyridine | pos | 108.0809 | M+H-H2O | C7H11NO | 9.6959 | 0.011382 |
| Xi-5-Hydroxydodecanoic acid | pos | 455.3358 | 2M+Na | C12H24O3 | 9.1501 | 0.011604 |
| 6-methyl-5-Hepten-2-one | pos | 127.1117 | M+H | C8H14O | 8.9199 | 0.009795 |
| MG(0:0/22:2(13Z,16Z)/0:0) | pos | 443.373 | M+CH3OH+H | C25H46O4 | 8.8575 | 0.011026 |
| DG(8:0/13:0/0:0) | pos | 459.3071 | M+2Na-H | C24H46O5 | 8.7721 | 0.010849 |
| Leontogenin | pos | 479.3335 | M+CH3OH+H | C27H42O5 | 8.6448 | 0.008124 |
| PC(18:0/0:0) | pos | 546.3522 | M+Na | C26H54NO7P | 8.6011 | 0.014945 |
| Lucidenic acid M | pos | 495.3283 | M+CH3OH+H | C27H42O6 | 8.5374 | 0.006736 |
| (Z)-9-Cycloheptadecen-1-one | pos | 268.2629 | M+NH4 | C17H30O | 8.5163 | 0.018557 |
| 2E,7-Octadienal | pos | 125.0961 | M+H | C8H12O | 8.474 | 0.009099 |
| 6-Ethyl-4-methyl-3E,5E,7E-decatriene | pos | 179.1792 | M+H | C13H22 | 8.3695 | 0.006243 |
| 5-Ethyl-3-methyl-2E,4E,6E-nonatriene | pos | 165.1636 | M+H | C12H20 | 8.3695 | 0.00837 |
| 2,2,6,6-Tetramethyl-4-piperidinone | pos | 156.1381 | M+H | C9H17NO | 8.3695 | 0.008776 |
| N-Cyclohexylformamide | pos | 128.1069 | M+H | C7H13NO | 8.3695 | 0.024746 |
| Cumene | pos | 121.1012 | M+H | C9H12 | 8.3695 | 0.018441 |
| Tetracosapentaenoic acid (24:5n-6) | pos | 376.318 | M+NH4 | C24H38O2 | 8.2199 | 0.022619 |
| 1-Hexacosene | pos | 382.44 | M+NH4 | C26H52 | 8.1148 | 0.007147 |
| 9-hydroxy-5Z-nonenoic acid | pos | 173.117 | M+H | C9H16O3 | 7.9874 | 0.008817 |
| 3,5,5-Trimethyl-2-cyclopenten-1-one | pos | 125.0961 | M+H | C8H12O | 7.9874 | 0.000599 |
| Methenamine | pos | 141.1132 | M+H | C6H12N4 | 7.9229 | 0.007992 |
| Lysyl-Valine | pos | 532.3837 | 2M+ACN+H | C11H23N3O3 | 7.2531 | 0.009621 |
| 2-(hydroxymethyl)-7-methoxy-2-methyl-3,4-dihydro-2H-1-benzopyran-4,5-diol | pos | 205.0857 | M+H-2H2O | C12H16O5 | 7.2112 | 0.014309 |
| Isokobusone | pos | 223.1689 | M+H | C14H22O2 | 7.1037 | 0.006972 |
| (Z)-7-Hexadecen-1,16-olide | pos | 270.2423 | M+NH4 | C16H28O2 | 7.0171 | 0.000979 |
| Sphinganine | pos | 302.3048 | M+H | C18H39NO2 | 6.5788 | 0.006713 |
| 1,20-Eicosanediol | pos | 359.2873 | M+2Na-H | C20H42O2 | 6.5576 | 0.013244 |
| Polyoxyethylene 40 monostearate | pos | 346.331 | M+NH4 | C20H40O3 | 6.5576 | 0.00336 |
| 7,8-Dihydropteroic acid | pos | 279.101 | M+H-2H2O | C14H14N6O3 | 6.5363 | 0.001924 |
| (R)-Pabulenol | pos | 251.0698 | M+H-2H2O | C16H14O5 | 6.5363 | 0.00689 |
| 2-Indanone | pos | 133.0647 | M+H | C9H8O | 6.3239 | 0.007399 |
| Pectachol | pos | 460.2685 | M+NH4 | C26H34O6 | 6.1785 | 0.056868 |
| Austalide L | pos | 446.2529 | M+NH4 | C25H32O6 | 6.1785 | 0.007825 |
| Alpha-Methylstyrene | pos | 119.0856 | M+H | C9H10 | 6.1785 | 0.014158 |
| M-Xylene | pos | 107.0857 | M+H | C8H10 | 6.1785 | 0.027785 |
| Nandrolone | pos | 292.226 | M+NH4 | C18H26O2 | 6.0723 | 0.003029 |
| Xestoaminol C | pos | 230.2474 | M+H | C14H31NO | 5.9218 | 0.00808 |
| 6-Hydroxyoctadecanoic acid | pos | 318.2996 | M+NH4 | C18H36O3 | 5.7952 | 0.004083 |
| C16 Sphinganine | pos | 274.2735 | M+H | C16H35NO2 | 5.7952 | 0.004564 |
| 10,20-Dihydroxyeicosanoic acid | pos | 362.3257 | M+NH4 | C20H40O4 | 5.7747 | 0.007049 |
| Clausarinol | pos | 432.2373 | M+NH4 | C24H30O6 | 5.7543 | 0.058258 |
| Toluene | pos | 93.07024 | M+H | C7H8 | 5.6099 | 0.006905 |
| Goshuyic acid | pos | 242.211 | M+NH4 | C14H24O2 | 5.4854 | 0.010275 |
| (E)-4,8-Dimethyl-1,3,7-nonatriene | pos | 151.1479 | M+H | C11H18 | 5.4854 | 0.006056 |
| 11-Hydroxy-9-tridecenoic acid | pos | 229.1795 | M+H | C13H24O3 | 5.4651 | 0.006102 |
| 4-Vinylcyclohexene | pos | 109.1013 | M+H | C8H12 | 5.4854 | 0.021313 |
| Delta-Hexalactone | pos | 115.0754 | M+H | C6H10O2 | 5.3803 | 0.013925 |
| Rimantadine | pos | 180.1745 | M+H | C12H21N | 5.1445 | 0.006088 |
| 16-Hydroxy hexadecanoic acid | pos | 290.2683 | M+NH4 | C16H32O3 | 4.9244 | 0.005373 |
| 1-Hexanol | pos | 246.2423 | 2M+ACN+H | C6H14O | 4.9244 | 0.004206 |
| Yuzu lactone | pos | 214.1798 | M+NH4 | C12H20O2 | 4.4628 | 0.013478 |
| (8alpha,10beta,11beta)-3-Hydroxy-4,15-dinor-1(5)-xanthen-12,8-olide | pos | 242.1745 | M+NH4 | C13H20O3 | 4.1775 | 0.005708 |
| 3-methyl-2-Quinoxalinone | pos | 161.0707 | M+H | C9H8N2O | 3.5141 | 0.022708 |
| N-Acetylserotonin | pos | 201.1019 | M+H-H2O | C12H14N2O2 | 3.3138 | 0.017763 |
| Naphthalene epoxide | pos | 162.0911 | M+NH4 | C10H8O | 3.1134 | 0.008203 |
| 2-Oxo-1,2-dihydroquinoline-4-carboxylate | pos | 190.0497 | M+H | C10H7NO3 | 2.9819 | 0.00475 |
| Val Pro Pro Phe | pos | 459.2593 | M+H | C24H34N4O5 | 2.9171 | 0.013369 |
| Oxalosuccinic acid | pos | 223.0453 | M+CH3OH+H | C6H6O7 | 2.8514 | 0.004969 |
| Cysteinyl-Glutamine | pos | 214.0625 | M+H-2H2O | C8H15N3O4S | 2.6279 | 0.005864 |
| L-Methionine | pos | 191.0847 | M+ACN+H | C5H11NO2S | 2.5828 | 0.004218 |
| Pro Phe Pro | pos | 360.191 | M+H | C19H25N3O4 | 2.4514 | 0.009363 |
| Entacapone | pos | 270.0885 | M+H-2H2O | C14H15N3O5 | 2.4071 | 0.044848 |
| Norketamine | pos | 224.0833 | M+H | C12H14ClNO | 2.4071 | 0.029487 |
| Val Asp Glu Ile | pos | 475.239 | M+H | C20H34N4O9 | 2.34 | 0.014141 |
| 5-Hydroxyindoleacetic acid | pos | 174.0548 | M+H-H2O | C10H9NO3 | 2.2517 | 0.025931 |
| Pro Val Ser Glu | pos | 431.2128 | M+H | C18H30N4O8 | 2.187 | 0.000879 |
| Vinylacetylglycine | pos | 144.0654 | M+H | C6H9NO3 | 2.187 | 0.017381 |
| Cappariloside A | pos | 376.1531 | M+ACN+H | C16H18N2O6 | 2.0821 | 0.010683 |
| Nicotyrine | pos | 159.0913 | M+H | C10H10N2 | 2.0642 | 0.002809 |
| 6-Methylquinoline | pos | 144.0806 | M+H | C10H9N | 2.0642 | 0.007065 |
| 3-Methylindole | pos | 132.0807 | M+H | C9H9N | 2.0642 | 0.004003 |
| Bk-DMBDB | pos | 236.1277 | M+H | C13H17NO3 | 2.0021 | 0.013567 |
| 1,8-Diazacyclotetradecane-2,9-dione | pos | 227.1749 | M+H | C12H22N2O2 | 2.0021 | 0.011857 |
| Tryptophyl-Alanine | pos | 293.1602 | M+NH4 | C14H17N3O3 | 1.96 | 0.013482 |
| Glu Gln | pos | 276.1185 | M+H | C10H17N3O6 | 1.96 | 0.018698 |
| Aprobarbital | pos | 211.1074 | M+H | C10H14N2O3 | 1.9378 | 0.011993 |
| 5'-Deoxy-5'-(methylthio)adenosine | pos | 298.0963 | M+H | C11H15N5O3S | 1.9164 | 0.013929 |
| Benzylazanium | pos | 108.0809 | M+H | C7H9N | 1.8533 | 0.00992 |
| Toxoflavine | pos | 194.0671 | M+H | C7H7N5O2 | 1.8343 | 0.007119 |
| Alpha-Trisaccharide | pos | 516.2295 | M+H | C20H37NO14 | 1.8165 | 0.014276 |
| Isoleucyl-Lysine | pos | 242.1858 | M+H-H2O | C12H25N3O3 | 1.8165 | 0.018379 |
| Prolyl-Valine | pos | 197.1282 | M+H-H2O | C10H18N2O3 | 1.8165 | 0.008513 |
| 2-Phenylacetamide | pos | 136.0755 | M+H | C8H9NO | 1.8165 | 0.002564 |
| (S)-Pterosin D | pos | 271.1283 | M+Na | C15H20O3 | 1.7963 | 0.031849 |
| Dextrorphan O-glucuronide | pos | 456.1966 | M+Na | C23H31NO7 | 1.7747 | 0.015585 |
| L-2-Amino-3-methylenehexanoic acid | pos | 185.1282 | M+ACN+H | C7H13NO2 | 1.7747 | 0.007766 |
| Gly Ala Glu Ile | pos | 389.2022 | M+H | C16H28N4O7 | 1.7542 | 0.006635 |
| Methionyl-Serine | pos | 219.0793 | M+H-H2O | C8H16N2O4S | 1.7542 | 0.010011 |
| Val Pro | pos | 215.1387 | M+H | C10H18N2O3 | 1.7542 | 0.001901 |
| O-Ureidohomoserine | pos | 355.1605 | 2M+H | C5H11N3O4 | 1.7334 | 0.019058 |
| Alpha,beta-Didehydrotryptophan | pos | 203.0813 | M+H | C11H10N2O2 | 1.7334 | 0.008751 |
| 6-Dimethylaminopurine | pos | 164.0929 | M+H | C7H9N5 | 1.7334 | 0.043648 |
| Ala Gly Asp Pro | pos | 359.1553 | M+H | C14H22N4O7 | 1.713 | 0.000612 |
| Clavamycin E | pos | 288.1184 | M+H | C11H17N3O6 | 1.713 | 0.011303 |
| Xanthine | pos | 153.0404 | M+H | C5H4N4O2 | 1.713 | 0.004818 |
| Ser Pro Pro Glu | pos | 429.1973 | M+H | C18H28N4O8 | 1.6929 | 0.015799 |
| Arginyl-Proline | pos | 254.1606 | M+H-H2O | C11H21N5O3 | 1.6929 | 0.002849 |
| L-Isoleucine | pos | 132.1018 | M+H | C6H13NO2 | 1.6929 | 0.008059 |
| Guanosine | pos | 284.0984 | M+H | C10H13N5O5 | 1.6543 | 0.01223 |
| Vidarabine | pos | 268.1035 | M+H | C10H13N5O4 | 1.6543 | 0.016036 |
| 3-Sialyl-N-acetyllactosamine | pos | 657.234 | M+H-H2O | C25H42N2O19 | 1.6363 | 0.070475 |
| Histidinyl-Proline | pos | 235.1184 | M+H-H2O | C11H16N4O3 | 1.6363 | 0.004605 |
| Benzofuran | pos | 119.0492 | M+H | C8H6O | 1.6363 | 0.01788 |
| 4-Hydroxybenzaldehyde | pos | 123.0441 | M+H | C7H6O2 | 1.6363 | 0.013801 |
| Neuraminic acid | pos | 232.0822 | M+H-2H2O | C9H17NO8 | 1.6173 | 0.020809 |
| 3alpha,4,5,7alpha-Tetrahydro-5-hydroxy-1H-isoindole-1,3(2H)-dione | pos | 168.0653 | M+H | C8H9NO3 | 1.6173 | 0.01469 |
| 2-Pyrrolidinone | pos | 86.06047 | M+H | C4H7NO | 1.5535 | 0.048339 |
| Cysteinyl-Hydroxyproline | pos | 276.1009 | M+ACN+H | C8H14N2O4S | 1.2624 | 0.004441 |
| N-Nitrosoguvacoline | pos | 171.0762 | M+H | C7H10N2O3 | 1.1826 | 0.009079 |
| Gamma-Glutamyl-beta-(isoxazolin-5-on-2-yl)alanine | pos | 266.0743 | M+H-2H2O | C11H15N3O7 | 1.1436 | 0.010926 |
| L-Proline | pos | 116.0707 | M+H | C5H9NO2 | 1.1248 | 0.02162 |
| Cytidine 2'-phosphate | pos | 346.0404 | M+Na | C9H14N3O8P | 1.1038 | 0.004409 |
| Ala Ala Pro | pos | 258.1443 | M+H | C11H19N3O4 | 1.0829 | 0.015261 |
| Pro Arg | pos | 272.1711 | M+H | C11H21N5O3 | 1.0626 | 0.006991 |
| Thiomorpholine 3-carboxylate | pos | 148.0424 | M+H | C5H9NO2S | 0.8514 | 0.098012 |
| Cis-4-Carboxymethylenebut-2-en-4-olide | pos | 158.0446 | M+NH4 | C6H4O4 | 0.7941 | 0.042877 |
| Uracil | pos | 113.0347 | M+H | C4H4N2O2 | 0.7941 | 0.024184 |
| 3-Buten-1-amine | pos | 72.08141 | M+H | C4H9N | 0.7754 | 0.003247 |
| Methionyl-Hydroxyproline | pos | 263.1056 | M+H | C10H18N2O4S | 0.7398 | 0.006977 |
| 2-Aminoisobutyric acid | pos | 104.0708 | M+H | C4H9NO2 | 0.722 | 0.003446 |
| Adenine | pos | 136.0616 | M+H | C5H5N5 | 0.722 | 0.019262 |
| Niacinamide | pos | 123.0553 | M+H | C6H6N2O | 0.722 | 0.026366 |
| Cytosine | pos | 112.0507 | M+H | C4H5N3O | 0.722 | 0.042764 |
| 1-Pyrroline | pos | 70.06578 | M+H | C4H7N | 0.7038 | 0.008871 |
| Cys Glu Cys | pos | 354.0764 | M+H | C11H19N3O6S2 | 0.6839 | 0.00997 |
| Seryllysine | pos | 198.1234 | M+H-2H2O | C9H19N3O4 | 0.666 | 0.004369 |
| L-Prolinamide | pos | 115.0867 | M+H | C5H10N2O | 0.666 | 0.011567 |
| L-cis-3-Amino-2-pyrrolidinecarboxylic acid | pos | 113.0711 | M+H-H2O | C5H10N2O2 | 0.666 | 0.028038 |
| L-Lysine | pos | 147.1126 | M+H | C6H14N2O2 | 0.6293 | 0.002924 |
| Glycyl-glycine | pos | 177.0245 | M+2Na-H | C4H8N2O3 | 0.6108 | 0.005133 |
| 4-Pyrimidine Methanamine (hydrochloride) | pos | 110.0715 | M+H | C5H7N3 | 0.6108 | 0.024946 |
| 1-Butylamine | pos | 74.09706 | M+H | C4H11N | 0.0142 | 0.025111 |
| 2-Hydroxycinnamic acid | pos | 182.081 | M+H, M+NH4 | C9H8O3 | 1.6363 | 0.004491 |
| Allysine | pos | 187.1075 | M+H-H2O, M+ACN+H | C6H11NO3 | 0.7398 | 0.008034 |
| Choline | pos | 104.1072 | M+H | C5H13NO | 0.6293 | 0.018507 |
| Dodecanedioic acid | pos | 231.1586 | M+H | C12H22O4 | 9.9677 | 0.02024 |
| Dodecanoic acid | pos | 218.2111 | M+NH4 | C12H24O2 | 3.7801 | 0.008253 |
| Gamma-Terpinene | pos | 137.1323 | M+H | C10H16 | 4.3752 | 0.01258 |
| Indole | pos | 118.0652 | M+H | C8H7N | 2.1018 | 0.00368 |
| Kanamycin | pos | 548.2555 | M+ACN+Na | C18H36N4O11 | 1.7747 | 0.012885 |
| L-Glutamate | pos | 130.0498 | M+H-H2O, M+H | C5H9NO4 | 0.6839 | 0.007328 |
| N'-Formylkynurenine | pos | 219.076 | M+H-H2O | C11H12N2O4 | 2.208 | 0.005774 |
| N-Acetylneuraminic acid | pos | 274.0915 | M+H-2H2O, M+H, M+H-H2O | C11H19NO9 | 1.0626 | 0.001628 |
| Styrene | pos | 105.0701 | M+H | C8H8 | 5.6099 | 0.030098 |
| L-4-Hydroxyglutamate semialdehyde | neg | 146.0447 | M-H, M+Na-2H, M-H2O-H | C5H9NO4 | 0.6429 | 0.009848 |
| Fenugreekine | neg | 662.1023 | M-H, M+Cl, M+Na-2H | C21H27N7O14P2 | 1.5148 | 0.004683 |
| Glutaminylproline | neg | 242.1142 | M-H, M+FA-H, M-H2O-H | C10H17N3O4 | 0.7437 | 0.035856 |
| Porphobilinogen | neg | 225.0875 | M-H, M+FA-H, M-H2O-H | C10H14N2O4 | 1.8697 | 0.022891 |
| Sesaminol glucosyl-(1->2)-[glucosyl-(1->6)]-glucoside | neg | 427.1238 | M-2H, M-H, M+Na-2H | C38H48O22 | 1.8697 | 0.050585 |
| Valyl-Proline | neg | 213.1238 | M-H, M+FA-H, M-H2O-H | C10H18N2O3 | 1.8697 | 0.006756 |
| N5-Acetyl-N2-gamma-L-glutamyl-L-ornithine | neg | 302.1357 | M-H, M+Na-2H, M-H2O-H | C12H21N3O6 | 2.1157 | 0.004708 |
| DTDP | neg | 401.0155 | M-H, M+Na-2H, M+FA-H | C10H16N2O11P2 | 1.2228 | 0.034725 |
| Arabinofuranose | neg | 195.0502 | M+Na-2H, M+FA-H | C5H10O5 | 0.6637 | 0.011661 |
| D-Alanyl-D-alanine | neg | 159.0763 | M-H, M+FA-H | C6H12N2O3 | 0.704 | 0.022512 |
| Gluconolactone | neg | 177.0394 | M-H2O-H, M-H | C6H10O6 | 0.704 | 0.029651 |
| N-Acetylasparagine | neg | 173.0557 | M-H, M-H2O-H | C6H10N2O4 | 0.7231 | 0.028583 |
| N-(gamma-Glutamyl)ethanolamine | neg | 171.0765 | M-H2O-H, M+Na-2H | C7H14N2O4 | 0.7437 | 0.018386 |
| Glutamylproline | neg | 243.0982 | M-H, M+K-2H | C10H16N2O5 | 0.7627 | 0.011579 |
| 1-(Hydroxymethyl)-5,5-dimethyl-2,4-imidazolidinedione | neg | 203.0665 | M+FA-H, 2M+Hac-H | C6H10N2O3 | 0.7437 | 0.031114 |
| Uridine 5'-diphosphate | neg | 402.9949 | M-H, M+Na-2H | C9H14N2O12P2 | 0.8239 | 0.005866 |
| Aspartyl-Isoleucine | neg | 227.1032 | M-H2O-H, M-H | C10H18N2O5 | 2.0492 | 0.001372 |
| L-beta-aspartyl-L-phenylalanine | neg | 261.0879 | M-H2O-H, M-H | C13H16N2O5 | 2.1589 | 0.014081 |
| 6-Oxopiperidine-2-carboxylic acid | neg | 142.0497 | M-H, 2M+FA-H | C6H9NO3 | 2.137 | 0.008772 |
| Ethyl vanillin | neg | 165.0545 | M-H2O-H, M-H | C9H10O3 | 3.1968 | 0.004726 |
| Adenosine diphosphate ribose | neg | 540.0539 | M-H2O-H, M-H | C15H23N5O14P2 | 0.7231 | 0.006879 |
| Pseudouridine 5'-phosphate | neg | 323.0285 | M-H, M+Na-2H | C9H13N2O9P | 1.0459 | 0.011393 |
| Glycylproline | neg | 171.0765 | M-H, 2M-H | C7H12N2O3 | 1.1124 | 0.004091 |
| Cytidine monophosphate N-acetylneuraminic acid | neg | 613.1402 | M-H, M+Na-2H | C20H31N4O16P | 1.1124 | 0.011372 |
| LysoPC(16:0) | neg | 540.3308 | M+Cl, M+FA-H | C24H50NO7P | 7.972 | 0.020728 |
| LysoPC(18:1(9Z)) | neg | 566.3464 | M+Cl, M+FA-H | C26H52NO7P | 8.1988 | 0.020905 |
| N-Acetyl-L-aspartic acid | neg | 174.0397 | M-H2O-H, M-H | C6H9NO5 | 1.2005 | 0.004841 |
| Petroselinic acid | neg | 303.2328 | M-H, M+Na-2H | C18H34O2 | 8.7421 | 0.025447 |
| Glutamylserine | neg | 233.0774 | M-H2O-H, M-H | C8H14N2O6 | 0.7231 | 0.008556 |
| Guanosine monophosphate | neg | 362.0507 | M-H, M+Na-2H | C10H14N5O8P | 1.2439 | 0.010302 |
| Deoxythymidine diphosphate-l-rhamnose | neg | 547.0738 | M-H, M+Na-2H | C16H26N2O15P2 | 1.2439 | 0.004677 |
| Aspartyl-L-proline | neg | 229.0824 | M-H, M+Na-2H | C9H14N2O5 | 1.2659 | 0.005595 |
| Xanthylic acid | neg | 363.0348 | M-H, M+Na-2H | C10H13N4O9P | 1.3104 | 0.012565 |
| UDP-N-acetylmuraminate | neg | 678.0957 | M-2H, M-H | C20H31N3O19P2 | 1.5378 | 0.017926 |
| Glutamylalanine | neg | 199.0716 | M-H2O-H, 2M+Hac-H | C8H14N2O5 | 1.5607 | 0.014494 |
| 6,8-Dihydroxypurine | neg | 151.0249 | M-H2O-H, M-H | C5H4N4O2 | 1.6064 | 0.00509 |
| 8-Oxo-dGMP | neg | 725.098 | M-H2O-H, 2M-H | C10H14N5O8P | 1.6064 | 0.009985 |
| Lycoperdic acid | neg | 216.0507 | M-H2O-H, M-H | C8H11NO6 | 1.7607 | 0.006341 |
| (2S,3'S)-alpha-Amino-2-carboxy-5-oxo-1-pyrrolidinebutanoic acid | neg | 211.0718 | M-H2O-H, 2M-H | C9H14N2O5 | 1.7829 | 0.01167 |
| (gamma-Glutamyl-gamma-glutamyl)-S-methylcysteine | neg | 392.1133 | M-H2O-H, M-H | C14H23N3O8S | 1.7829 | 0.01475 |
| Aspartyl-Methionine | neg | 245.0599 | M-H2O-H, M-H | C9H16N2O5S | 1.7829 | 0.005314 |
| Methionyl-Alanine | neg | 219.0768 | M-H2O-H, M-H | C8H16N2O3S | 1.7829 | 0.005432 |
| Bromocriptine | neg | 652.2114 | M-H, M+Na-2H | C32H40BrN5O5 | 1.8267 | 0.012608 |
| Acetyl-CoA | neg | 403.5556 | M-2H, M-H | C23H38N7O17P3S | 1.8916 | 0.023431 |
| Sedoheptulose 1,7-bisphosphate | neg | 368.9993 | M-H, M+Na-2H | C7H16O13P2 | 0.5426 | 0.016777 |
| 2-(3-Carboxy-3-aminopropyl)-L-histidine | neg | 301.1152 | M+FA-H | C10H16N4O4 | 0.6637 | 0.005271 |
| L-beta-aspartyl-L-alanine | neg | 203.0665 | M-H | C7H12N2O5 | 1.0678 | 0.006812 |
| FAPy-adenine | neg | 134.0459 | M-H2O-H | C5H7N5O | 1.1341 | 0.022999 |
| DCMP | neg | 306.0497 | M-H | C9H14N3O7P | 1.1341 | 0.020461 |
| Asparaginyl-Proline | neg | 228.0984 | M-H | C9H15N3O4 | 1.1782 | 0.005867 |
| (2R,2'S)-Isobuteine | neg | 206.0484 | M-H | C7H13NO4S | 1.3556 | 0.004746 |
| Hypoxanthine | neg | 135.0299 | M-H | C5H4N4O | 1.4237 | 0.024363 |
| Threoninyl-Proline | neg | 215.103 | M-H | C9H16N2O4 | 1.5378 | 0.019876 |
| D-4'-Phosphopantothenate | neg | 298.0695 | M-H | C9H18NO8P | 1.5834 | 0.010963 |
| Blasticidin S | neg | 403.1833 | M-H2O-H | C17H26N8O5 | 1.5834 | 0.014782 |
| L-prolyl-L-proline | neg | 211.1081 | M-H | C10H16N2O3 | 1.7386 | 0.020996 |
| Glucosylgalactosyl hydroxylysine | neg | 521.1737 | M+Cl | C18H34N2O13 | 1.7386 | 0.024976 |
| N-(3-acetamidopropyl)pyrrolidin-2-one | neg | 229.1188 | M+FA-H | C9H16N2O2 | 1.7607 | 0.007992 |
| Gamma-Glutamylglutamic acid | neg | 257.0777 | M-H2O-H | C10H16N2O7 | 1.7607 | 0.00663 |
| Thymidine | neg | 287.0885 | M+FA-H | C10H14N2O5 | 1.7607 | 0.012753 |
| Glucosyl 6-hydroxy-2,6-dimethyl-2E,7-octadienoate | neg | 381.1337 | M+Cl | C16H26O8 | 1.7607 | 0.007521 |
| 6-Hydroxymelatonin glucuronide | neg | 459.1209 | M+Cl | C19H24N2O9 | 1.7607 | 0.031071 |
| Telmisartan | neg | 513.2315 | M-H | C33H30N4O2 | 1.8484 | 0.023211 |
| Tyrosyl-Proline | neg | 277.1192 | M-H | C14H18N2O4 | 1.8697 | 0.031281 |
| FAD | neg | 784.1505 | M-H | C27H33N9O15P2 | 1.8916 | 0.00498 |
| Isoleucylproline | neg | 227.1396 | M-H | C11H20N2O3 | 1.9812 | 0.021178 |
| Coumarin | neg | 181.0052 | M+Cl | C9H6O2 | 2.071 | 0.00589 |
| Olopatadine n-oxide | neg | 374.1392 | M+Na-2H | C21H23NO4 | 2.0934 | 0.005584 |
| N-Acetyl-L-methionine | neg | 190.0534 | M-H | C7H13NO3S | 2.137 | 0.003322 |
| Gamma-Glutamylisoleucine | neg | 259.1298 | M-H | C11H20N2O5 | 2.2498 | 0.010553 |
| Pirbuterol | neg | 285.1455 | M+FA-H | C12H20N2O3 | 2.7501 | 0.020144 |
| Glutamylisoleucine | neg | 241.119 | M-H2O-H | C11H20N2O5 | 2.7957 | 0.020226 |
| N-Acetylleucine | neg | 172.0968 | M-H | C8H15NO3 | 3.0854 | 0.004675 |
| 5,7-dihydroxy-2-(4-hydroxyphenyl)-8-(3,4,5-trihydroxyoxan-2-yl)-4H-chromen-4-one | neg | 401.0877 | M-H | C20H18O9 | 4.7327 | 0.017167 |
| Neocnidilide | neg | 239.1284 | M+FA-H | C12H18O2 | 6.2258 | 0.014121 |
| 13S-hydroxyoctadecadienoic acid | neg | 295.2276 | M-H | C18H32O3 | 7.3932 | 0.015368 |
| Cavipetin C | neg | 409.236 | M+Na-2H | C24H36O4 | 8.3911 | 0.015576 |
| Tiglylcarnitine | neg | 485.2825 | 2M-H | C12H21NO4 | 8.7421 | 0.023225 |
| 1-Stearoylglycerophosphoglycerol | neg | 511.3039 | M-H | C24H49O9P | 8.9313 | 0.014305 |
| Retinyl beta-glucuronide | neg | 443.2473 | M-H2O-H | C26H38O7 | 9.5588 | 0.004437 |
| 4alpha-carboxy-5alpha-cholesta-8,24-dien-3beta-ol | neg | 409.311 | M-H2O-H | C28H44O3 | 9.5773 | 0.004405 |
| Manglupenone | neg | 473.2823 | M+K-2H | C30H44O2 | 9.1883 | 0.004471 |
| Stearaldehyde | neg | 313.2748 | M+FA-H | C18H36O | 8.876 | 0.028502 |
| MG(19:0/0:0/0:0) | neg | 371.3165 | M-H | C22H44O4 | 8.3686 | 0.015613 |
| 2-Oleoylglycerophosphocholine | neg | 557.3208 | M+Cl | C26H53NO7P+ | 7.972 | 0.021669 |
| LysoPC(15:0) | neg | 480.3096 | M-H | C23H48NO7P | 7.972 | 0.017803 |
| 2-Dodecylbenzenesulfonic acid | neg | 325.1841 | M-H | C18H30O3S | 7.8806 | 0.01757 |
| (9S,10S)-9,10-dihydroxyoctadecanoate | neg | 315.2538 | M-H | C18H36O4 | 7.202 | 0.025726 |
| (S)-Bilobanone | neg | 277.1443 | M+FA-H | C15H20O2 | 7.004 | 0.017388 |
| 5-Nonadecyl-1,3-benzenediol | neg | 411.3053 | M+Cl | C25H44O2 | 6.5499 | 0.014301 |
| 12-Oxo-2,3-dinor-10,15-phytodienoic acid | neg | 309.1707 | M+FA-H | C16H24O3 | 6.3831 | 0.019522 |
| Furanofukinin | neg | 293.1758 | M+FA-H | C16H24O2 | 6.3397 | 0.019033 |
| 3-Hydroxypicolinic acid | neg | 138.0184 | M-H | C6H5NO3 | 3.982 | 0.023511 |
| Shoyuflavone A | neg | 421.0349 | M+Cl | C19H14O9 | 3.2189 | 0.038811 |
| N-Acetyl-L-phenylalanine | neg | 206.0814 | M-H | C11H13NO3 | 3.2189 | 0.015559 |
| Stavudine | neg | 261.029 | M+K-2H | C10H12N2O4 | 3.1524 | 0.006755 |
| (2E)-3-(2,4-dihydroxy-5-methoxyphenyl)prop-2-enoic acid | neg | 209.0448 | M-H | C10H10O5 | 2.9969 | 0.005052 |
| Succinylacetone | neg | 157.0495 | M-H | C7H10O4 | 2.8412 | 0.011239 |
| 3-(3,4,5-Trimethoxyphenyl)propanoic acid | neg | 239.0921 | M-H | C12H16O5 | 2.7957 | 0.04378 |
| 5-Phenyl-1,3-oxazinane-2,4-dione | neg | 172.0394 | M-H2O-H | C10H9NO3 | 2.5344 | 0.021828 |
| Glutamylphenylalanine | neg | 275.1038 | M-H2O-H | C14H18N2O5 | 2.4923 | 0.004907 |
| Phenylalanylproline | neg | 261.1243 | M-H | C14H18N2O3 | 2.473 | 0.00723 |
| Hypoglycin B | neg | 315.1199 | M+FA-H | C12H18N2O5 | 2.4097 | 0.017919 |
| Gamma-Glutamylphenylalanine | neg | 293.1145 | M-H | C14H18N2O5 | 2.3641 | 0.006816 |
| Cinncassiol A | neg | 403.1723 | M+Na-2H | C20H30O7 | 2.2955 | 0.020876 |
| N-Acetyl-L-tyrosine | neg | 222.0765 | M-H | C11H13NO4 | 2.0934 | 0.003021 |
| Lamivudine sulfoxide | neg | 266.02 | M+Na-2H | C8H11N3O4S | 2.071 | 0.003482 |
| L-Tryptophan | neg | 203.0817 | M-H | C11H12N2O2 | 2.0269 | 0.005805 |
| Dide-O-methylsimmondsin | neg | 392.1204 | M+FA-H | C14H21NO9 | 1.9812 | 0.014411 |
| Serylisoleucine | neg | 217.1187 | M-H | C9H18N2O4 | 1.9583 | 0.010496 |
| 2-Hydroxy-imipramine glucuronide | neg | 509.1652 | M+K-2H | C25H32N2O7 | 1.8916 | 0.010775 |
| N-(2-Hydroxyisobutyl)-2,4,8,10,12-tetradecapentaenamide | neg | 310.1773 | M+Na-2H | C18H27NO2 | 1.8916 | 0.005155 |
| 1alpha-Hydroxyarbusculin A | neg | 287.1249 | M+Na-2H | C15H22O4 | 1.8916 | 0.051285 |
| Pantothenic acid | neg | 218.1028 | M-H | C9H17NO5 | 1.8916 | 0.042969 |
| Benzyl glycinate | neg | 164.0706 | M-H | C9H11NO2 | 1.8916 | 0.0049 |
| 2,3-Butanediol apiosylglucoside | neg | 383.1572 | M-H | C15H28O11 | 1.8484 | 0.012333 |
| Gallic acid | neg | 169.0132 | M-H | C7H6O5 | 1.8047 | 0.072568 |
| 2-(Arabinosylamino)-3-(glucosylamino)propanenitrile | neg | 360.1413 | M-H2O-H | C14H25N3O9 | 1.7607 | 0.005887 |
| N-Succinyl-L,L-2,6-diaminopimelate | neg | 289.1041 | M-H | C11H18N2O7 | 1.7607 | 0.024069 |
| Deoxyinosine | neg | 251.0783 | M-H | C10H12N4O4 | 1.7386 | 0.009375 |
| 2-Pyrrolidineacetic acid | neg | 174.0761 | M+FA-H | C6H11NO2 | 1.7386 | 0.009102 |
| 2-Hydroxyadenine | neg | 150.0409 | M-H | C5H5N5O | 1.5834 | 0.203956 |
| L-Tyrosine | neg | 180.0656 | M-H | C9H11NO3 | 1.5607 | 0.019678 |
| N-Acetylglutamic acid | neg | 188.0555 | M-H | C7H11NO5 | 1.5607 | 0.003865 |
| {[(3E)-4-(1-oxo-1H-isochromen-3-yl)but-3-en-2-yl]oxy}sulfonic acid | neg | 637.0692 | 2M+FA-H | C13H12O6S | 1.5378 | 0.012052 |
| DTDP-D-glucose | neg | 545.0579 | M-H2O-H | C16H26N2O16P2 | 1.5378 | 0.049318 |
| Deoxyadenosine monophosphate | neg | 330.0609 | M-H | C10H14N5O6P | 1.5378 | 0.004803 |
| 5-Thymidylic acid | neg | 321.0492 | M-H | C10H15N2O8P | 1.5378 | 0.014792 |
| Oxoglutaric acid | neg | 191.0188 | M+FA-H | C5H6O5 | 1.333 | 0.009752 |
| Alanyl-Proline | neg | 185.0922 | M-H | C8H14N2O3 | 1.333 | 0.005251 |
| Gamma glutamyl ornithine | neg | 242.1142 | M-H2O-H | C10H19N3O5 | 1.2659 | 0.005363 |
| Citramalic acid | neg | 147.0287 | M-H | C5H8O5 | 1.2439 | 0.005167 |
| Gamma-Glutamyl-beta-cyanoalanine | neg | 242.0779 | M-H | C9H13N3O5 | 1.1341 | 0.004859 |
| Guanosine diphosphate | neg | 442.017 | M-H | C10H15N5O11P2 | 1.0903 | 0.014387 |
| Trans-S-(1-Propenyl)-L-cysteine | neg | 206.0485 | M+FA-H | C6H11NO2S | 0.7815 | 0.005021 |
| N-Acetyl-9-O-acetylneuraminic acid | neg | 350.1094 | M-H | C13H21NO10 | 0.7437 | 0.019082 |
| Hydroxyprolyl-Asparagine | neg | 290.0992 | M+FA-H | C9H15N3O5 | 0.704 | 0.009267 |
| L-Glutamine | neg | 145.0607 | M-H | C5H10N2O3 | 0.6637 | 0.010272 |
| 1D-Myo-inositol 3,4-bisphosphate | neg | 338.9887 | M-H | C6H14O12P2 | 0.5426 | 0.015411 |
| Adenosine 2'-phosphate | neg | 346.0556 | M-H, 2M-H | C10H14N5O7P | 1.2005 | 0.013263 |
| Cytidine monophosphate | neg | 322.0444 | M-H | C9H14N3O8P | 1.0678 | 0.010889 |
| D-Apiose | neg | 149.0443 | M-H | C5H10O5 | 0.704 | 0.029923 |
| D-Glucose | neg | 179.0551 | M-H2O-H, M-H | C6H12O6 | 0.6831 | 0.010264 |
| D-Ribose 5-phosphate | neg | 211.0006 | M-H2O-H | C5H11O8P | 0.7231 | 0.005522 |
| Glucosamine | neg | 160.0604 | M-H2O-H | C6H13NO5 | 1.333 | 0.009413 |
| Pentobarbital | neg | 271.1299 | M+FA-H | C11H18N2O3 | 2.0492 | 0.006694 |
