# Supplementary material for: Transcriptome and metabolome profiling to elucidate the mechanism underlying the poor growth of Streptococcus suis serotype 2 after orphan response regulator CovR deletion
Source: Front Vet Sci. 2023 Nov 7;10:1280161. doi: 10.3389/fvets.2023.1280161 (PMC10661955; doi:10.3389/fvets.2023.1280161)
Supplement: Supplementary file 3 [file Table_2.docx]

**Supplementary table S2** The list of 146 diferential accumulation metabolites detected in and mutant Δ*covR* strain compared to *S. suis* 2 SC19

| Metabolite | Regulate | Formula | VIP_pred_OPLS-DA | VIP_PLS-DA | FC (Δ*covR* /SC19) |
| --- | --- | --- | --- | --- | --- |
| Asn Pro | up | C9H15N3O4 | 1.4989 | 1.4888 | 1.0238 |
| 1-Aminocyclopropanecarboxylic acid | up | C4H7NO2 | 1.3443 | 1.3281 | 1.0182 |
| Citrulline | down | C6H13N3O3 | 1.0716 | 1.0668 | 0.9887 |
| Lactulose | down | C12H22O11 | 1.3832 | 1.3798 | 0.9788 |
| 3-Deoxy-D-glycero-D-galacto-2-nonulosonic acid | down | C9H16O9 | 1.9797 | 1.98 | 0.9508 |
| 1-heneicosanoyl-glycero-3-phosphate | up | C24H49O7P | 1.2753 | 1.2688 | 1.0179 |
| Gln Pro | up | C10H17N3O4 | 1.4735 | 1.4645 | 1.0201 |
| Threoninyl-Glutamate | up | C9H16N2O6 | 1.444 | 1.4443 | 1.0243 |
| Inosine | down | C10H12N4O5 | 2.0964 | 2.0881 | 0.9501 |
| Pro Leu | up | C11H20N2O3 | 2.3347 | 2.3268 | 1.0515 |
| (+/-)-2-Hydroxy-4-(methylthio)butanoic acid | down | C5H10O3S | 1.8986 | 1.899 | 0.9536 |
| L-Ornithine | down | C5H12N2O2 | 1.9042 | 1.9006 | 0.9571 |
| 4-Methylene-L-glutamine | down | C6H10N2O3 | 1.0443 | 1.03 | 0.9886 |
| Pro Pro | up | C10H16N2O3 | 1.0629 | 1.0674 | 1.0112 |
| D-Pipecolic acid | up | C6H11NO2 | 1.201 | 1.2021 | 1.016 |
| PC(18:2/0:0) | up | C26H50NO7P | 1.1949 | 1.181 | 1.0205 |
| PC(16:0/0:0) | up | C24H50NO7P | 1.2452 | 1.2396 | 1.0169 |
| 5'-CMP | up | C9H14N3O8P | 2.0883 | 2.0873 | 1.0458 |
| L-Aspartic Acid | down | C4H7NO4 | 2.5661 | 2.5451 | 0.9198 |
| N-acetylaspartate | down | C6H9NO5 | 2.7557 | 2.733 | 0.9047 |
| Aspartyl-Proline | down | C9H14N2O5 | 1.3842 | 1.3778 | 0.9789 |
| 4-Hydroxyproline galactoside | up | C11H19NO8 | 2.0121 | 1.9988 | 1.0461 |
| Thr Pro | up | C9H16N2O4 | 1.6639 | 1.6464 | 1.0287 |
| Tetrahydropentoxyline | down | C17H22N2O7 | 1.5534 | 1.5388 | 0.9681 |
| Gly Pro Val | up | C12H21N3O4 | 2.6912 | 2.674 | 1.0957 |
| Pro Ser Pro Pro | up | C18H28N4O6 | 1.2741 | 1.2614 | 1.0274 |
| Methyl 1-methoxy-1H-indole-3-carboxylate | up | C11H11NO3 | 2.3236 | 2.277 | 1.088 |
| Lumichrome | up | C12H10N4O2 | 1.8341 | 1.8108 | 1.0482 |
| 5-Ethyl-2,3-dimethylpyrazine | down | C8H12N2 | 1.8098 | 1.8211 | 0.9475 |
| Pro His | up | C11H16N4O3 | 1.5342 | 1.5331 | 1.0286 |
| Ser Pro | up | C8H14N2O4 | 1.7461 | 1.7411 | 1.0399 |
| Allobarbital | up | C10H12N2O3 | 1.5751 | 1.5696 | 1.0316 |
| Ala Pro | up | C8H14N2O3 | 1.8313 | 1.8204 | 1.0337 |
| Histidinyl-Isoleucine | up | C12H20N4O3 | 1.7138 | 1.7099 | 1.0514 |
| 4-Hydroxy-5-phenyltetrahydro-1,3-oxazin-2-one | up | C10H11NO3 | 1.5064 | 1.484 | 1.0454 |
| Val Pro Pro | up | C15H25N3O4 | 1.6801 | 1.6756 | 1.0268 |
| Gly Pro Leu | up | C13H23N3O4 | 2.0876 | 2.0889 | 1.0542 |
| 4-formyl Indole | up | C9H7NO | 1.0747 | 1.0539 | 1.0158 |
| L-beta-aspartyl-L-leucine | down | C10H18N2O5 | 1.0294 | 1.0268 | 0.9875 |
| Leucyl-Gamma-glutamate | up | C11H21N3O4 | 1.4781 | 1.4662 | 1.033 |
| L-phenylalanyl-L-proline | up | C14H18N2O3 | 2.1434 | 2.1347 | 1.0441 |
| (+/-)-2-Methylthiazolidine | down | C4H9NS | 1.7989 | 1.8057 | 0.9519 |
| Asp Pro Ile | up | C15H25N3O6 | 1.0208 | 1.0037 | 1.0159 |
| Gamma-Glu-Leu | down | C11H20N2O5 | 1.028 | 1.0165 | 0.9837 |
| Hydroxypentobarbital | up | C11H18N2O4 | 1.4028 | 1.3959 | 1.0286 |
| 3-Aminoquinoline | up | C9H8N2 | 1.9906 | 1.9591 | 1.064 |
| Hericene B | up | C37H58O5 | 1.4374 | 1.4377 | 1.0278 |
| Docosadienoate (22:2n6) | up | C22H40O2 | 1.0263 | 1.0253 | 1.0153 |
| 3-methyl-2-Quinoxalinone | up | C9H8N2O | 2.634 | 2.5871 | 1.1048 |
| Naphthalene epoxide | down | C10H8O | 1.0728 | 1.0908 | 0.9781 |
| 2-Oxo-1,2-dihydroquinoline-4-carboxylate | up | C10H7NO3 | 2.9437 | 2.8915 | 1.1552 |
| Val Pro Pro Phe | down | C24H34N4O5 | 1.7565 | 1.7712 | 0.9469 |
| Oxalosuccinic acid | up | C6H6O7 | 1.083 | 1.0802 | 1.0213 |
| L-Methionine | up | C5H11NO2S | 1.9654 | 1.9512 | 1.0663 |
| Pro Phe Pro | up | C19H25N3O4 | 1.9715 | 1.9631 | 1.0417 |
| Entacapone | down | C14H15N3O5 | 1.4399 | 1.4165 | 0.9739 |
| Norketamine | down | C12H14ClNO | 1.3762 | 1.3567 | 0.9709 |
| 5-Hydroxyindoleacetic acid | up | C10H9NO3 | 2.3847 | 2.3415 | 1.0852 |
| Vinylacetylglycine | down | C6H9NO3 | 1.7485 | 1.7541 | 0.9631 |
| Bk-DMBDB | down | C13H17NO3 | 1.0333 | 1.0178 | 0.9817 |
| Tryptophyl-Alanine | down | C14H17N3O3 | 1.6829 | 1.675 | 0.9639 |
| Prolyl-Valine | up | C10H18N2O3 | 1.6967 | 1.6896 | 1.0338 |
| (S)-Pterosin D | down | C15H20O3 | 1.9898 | 1.9571 | 0.9367 |
| Dextrorphan O-glucuronide | down | C23H31NO7 | 1.63 | 1.6105 | 0.9645 |
| Gly Ala Glu Ile | down | C16H28N4O7 | 1.2078 | 1.1697 | 0.9765 |
| Val Pro | up | C10H18N2O3 | 2.3308 | 2.3223 | 1.0523 |
| O-Ureidohomoserine | down | C5H11N3O4 | 1.3459 | 1.3128 | 0.9727 |
| Alpha,beta-Didehydrotryptophan | up | C11H10N2O2 | 1.5129 | 1.4973 | 1.0347 |
| Ala Gly Asp Pro | down | C14H22N4O7 | 1.2836 | 1.2582 | 0.9761 |
| Ser Pro Pro Glu | up | C18H28N4O8 | 1.1345 | 1.1443 | 1.0196 |
| Histidinyl-Proline | up | C11H16N4O3 | 1.6105 | 1.5877 | 1.0344 |
| 3alpha,4,5,7alpha-Tetrahydro-5-hydroxy-1H-isoindole-1,3(2H)-dione | up | C8H9NO3 | 1.4778 | 1.4641 | 1.0282 |
| 2-Pyrrolidinone | down | C4H7NO | 1.0059 | 0.9901 | 0.9843 |
| Cysteinyl-Hydroxyproline | up | C8H14N2O4S | 2.3621 | 2.3392 | 1.0727 |
| N-Nitrosoguvacoline | up | C7H10N2O3 | 1.5451 | 1.5379 | 1.0312 |
| Gamma-Glutamyl-beta-(isoxazolin-5-on-2-yl)alanine | down | C11H15N3O7 | 2.0935 | 2.0863 | 0.9441 |
| Cytidine 2'-phosphate | up | C9H14N3O8P | 2.0458 | 2.0442 | 1.0462 |
| Ala Ala Pro | up | C11H19N3O4 | 1.9206 | 1.9066 | 1.0489 |
| Pro Arg | up | C11H21N5O3 | 1.6678 | 1.6581 | 1.0382 |
| Cis-4-Carboxymethylenebut-2-en-4-olide | down | C6H4O4 | 2.5591 | 2.5341 | 0.918 |
| Methionyl-Hydroxyproline | up | C10H18N2O4S | 1.5673 | 1.5525 | 1.0306 |
| Cys Glu Cys | up | C11H19N3O6S2 | 1.883 | 1.875 | 1.0425 |
| Seryllysine | down | C9H19N3O4 | 2.0745 | 2.0816 | 0.9486 |
| L-Prolinamide | down | C5H10N2O | 1.662 | 1.655 | 0.9674 |
| L-Lysine | up | C6H14N2O2 | 1.8628 | 1.8582 | 1.0416 |
| Allysine | up | C6H11NO3 | 1.8137 | 1.8003 | 1.0349 |
| Kanamycin | down | C18H36N4O11 | 1.1996 | 1.1711 | 0.9803 |
| L-Glutamate | down | C5H9NO4 | 1.1714 | 1.1613 | 0.9861 |
| N'-Formylkynurenine | up | C11H12N2O4 | 2.499 | 2.4547 | 1.1034 |
| N-Acetylneuraminic acid | up | C11H19NO9 | 2.0776 | 2.0798 | 1.0466 |
| Glutaminylproline | up | C10H17N3O4 | 1.7177 | 1.7096 | 1.0361 |
| Sesaminol glucosyl-(1->2)-[glucosyl-(1->6)]-glucoside | up | C38H48O22 | 1.7809 | 1.7667 | 1.0381 |
| Valyl-Proline | up | C10H18N2O3 | 2.5138 | 2.5003 | 1.0749 |
| Gluconolactone | down | C6H10O6 | 1.0441 | 1.0642 | 0.9819 |
| N-Acetylasparagine | down | C6H10N2O4 | 2.3733 | 2.3472 | 0.9311 |
| Uridine 5'-diphosphate | down | C9H14N2O12P2 | 1.0366 | 1.0145 | 0.988 |
| 6-Oxopiperidine-2-carboxylic acid | down | C6H9NO3 | 1.67 | 1.6736 | 0.962 |
| Pseudouridine 5'-phosphate | down | C9H13N2O9P | 1.0534 | 1.046 | 0.9868 |
| Cytidine monophosphate N-acetylneuraminic acid | up | C20H31N4O16P | 2.072 | 2.0661 | 1.0436 |
| LysoPC(16:0) | up | C24H50NO7P | 1.331 | 1.3166 | 1.0205 |
| N-Acetyl-L-aspartic acid | down | C6H9NO5 | 2.4442 | 2.4307 | 0.9397 |
| Deoxythymidine diphosphate-l-rhamnose | down | C16H26N2O15P2 | 1.0019 | 0.998 | 0.9891 |
| Aspartyl-L-proline | down | C9H14N2O5 | 1.3612 | 1.3678 | 0.9748 |
| Xanthylic acid | down | C10H13N4O9P | 1.8189 | 1.8042 | 0.962 |
| 6,8-Dihydroxypurine | up | C5H4N4O2 | 1.349 | 1.3543 | 1.0352 |
| Lycoperdic acid | down | C8H11NO6 | 1.3113 | 1.309 | 0.9766 |
| Methionyl-Alanine | up | C8H16N2O3S | 1.4671 | 1.4425 | 1.0334 |
| Acetyl-CoA | up | C23H38N7O17P3S | 1.973 | 1.9776 | 1.0654 |
| Asparaginyl-Proline | up | C9H15N3O4 | 1.6188 | 1.6031 | 1.0349 |
| Hypoxanthine | down | C5H4N4O | 1.4845 | 1.4648 | 0.9764 |
| Threoninyl-Proline | up | C9H16N2O4 | 1.6249 | 1.63 | 1.0338 |
| D-4'-Phosphopantothenate | down | C9H18NO8P | 2.132 | 2.152 | 0.9485 |
| L-prolyl-L-proline | up | C10H16N2O3 | 1.1083 | 1.0909 | 1.0199 |
| Thymidine | down | C10H14N2O5 | 1.4257 | 1.4292 | 0.9706 |
| Glucosyl 6-hydroxy-2,6-dimethyl-2E,7-octadienoate | down | C16H26O8 | 1.2267 | 1.2401 | 0.9706 |
| Tyrosyl-Proline | up | C14H18N2O4 | 1.387 | 1.4045 | 1.0244 |
| Isoleucylproline | up | C11H20N2O3 | 2.5255 | 2.507 | 1.0745 |
| N-Acetyl-L-methionine | down | C7H13NO3S | 2.0015 | 2.0077 | 0.9533 |
| Pirbuterol | up | C12H20N2O3 | 1.6216 | 1.6091 | 1.0379 |
| Glutamylisoleucine | up | C11H20N2O5 | 1.3335 | 1.327 | 1.0261 |
| N-Acetylleucine | down | C8H15NO3 | 1.0955 | 1.1012 | 0.9802 |
| Cavipetin C | up | C24H36O4 | 1.3923 | 1.3637 | 1.0289 |
| 1-Stearoylglycerophosphoglycerol | up | C24H49O9P | 1.7904 | 1.7896 | 1.0395 |
| 4alpha-carboxy-5alpha-cholesta-8,24-dien-3beta-ol | up | C28H44O3 | 1.4589 | 1.4208 | 1.0373 |
| LysoPC(15:0) | up | C23H48NO7P | 1.3009 | 1.2874 | 1.0244 |
| (S)-Bilobanone | down | C15H20O2 | 1.0799 | 1.0864 | 0.9816 |
| N-Acetyl-L-phenylalanine | down | C11H13NO3 | 1.4523 | 1.4643 | 0.9687 |
| 3-(3,4,5-Trimethoxyphenyl)propanoic acid | down | C12H16O5 | 1.7473 | 1.7535 | 0.951 |
| 5-Phenyl-1,3-oxazinane-2,4-dione | up | C10H9NO3 | 2.1377 | 2.0963 | 1.0802 |
| Phenylalanylproline | up | C14H18N2O3 | 2.1323 | 2.1188 | 1.0519 |
| Hypoglycin B | down | C12H18N2O5 | 1.3415 | 1.3435 | 0.9797 |
| Gamma-Glutamylphenylalanine | down | C14H18N2O5 | 1.1437 | 1.1476 | 0.9806 |
| N-Acetyl-L-tyrosine | down | C11H13NO4 | 1.4128 | 1.4103 | 0.9695 |
| 2-Hydroxy-imipramine glucuronide | down | C25H32N2O7 | 1.5381 | 1.5483 | 0.9621 |
| N-(2-Hydroxyisobutyl)-2,4,8,10,12-tetradecapentaenamide | up | C18H27NO2 | 1.8291 | 1.8106 | 1.0434 |
| 2-(Arabinosylamino)-3-(glucosylamino)propanenitrile | down | C14H25N3O9 | 1.0379 | 1.0398 | 0.9848 |
| Deoxyinosine | down | C10H12N4O4 | 1.8723 | 1.9021 | 0.943 |
| N-Acetylglutamic acid | down | C7H11NO5 | 2.0834 | 2.0438 | 0.9351 |
| Alanyl-Proline | up | C8H14N2O3 | 1.891 | 1.8929 | 1.0457 |
| Gamma glutamyl ornithine | up | C10H19N3O5 | 1.5496 | 1.548 | 1.0278 |
| Citramalic acid | up | C5H8O5 | 2.1962 | 2.1883 | 1.073 |
| Gamma-Glutamyl-beta-cyanoalanine | down | C9H13N3O5 | 1.4573 | 1.4546 | 0.9696 |
| N-Acetyl-9-O-acetylneuraminic acid | up | C13H21NO10 | 2.1833 | 2.1758 | 1.0564 |
| L-Glutamine | down | C5H10N2O3 | 1.297 | 1.3175 | 0.9749 |
| Cytidine monophosphate | up | C9H14N3O8P | 1.8677 | 1.8647 | 1.036 |
| Glucosamine | down | C6H13NO5 | 1.0673 | 1.0491 | 0.9831 |
