# Supplementary material for: Transcriptome and metabolome profiling to elucidate the mechanism underlying the poor growth of Streptococcus suis serotype 2 after orphan response regulator CovR deletion
Source: Front Vet Sci. 2023 Nov 7;10:1280161. doi: 10.3389/fvets.2023.1280161 (PMC10661955; doi:10.3389/fvets.2023.1280161)
Supplement: Supplementary file 4 [file Table_3.docx]

**Supplementary table S3** The differentially expressed genes of 5 KEGG enrichment pathways detected in the Δ*covR* compared to SC19

| Pathways  （p_value） | Gene ID | Ko name | Gene description | FC (ΔcovR/SC19) | *P*_value |
| --- | --- | --- | --- | --- | --- |
| ABC transporters  (<5e-324) | B9H01_RS09035 | lysX1 | ABC transporter substrate-binding protein/permease | 1.924 | 4.80E-29 |
|  | B9H01_RS06570 | peb1C | amino acid ABC transporter ATP-binding protein | 1.919 | 2.77E-06 |
|  | B9H01_RS06575 | peb1A | transporter substrate-binding domain-containing protein | 2.001 | 6.75E-11 |
|  | B9H01_RS04995 | artP | transporter substrate-binding domain-containing protein | 2.025 | 0.001155 |
|  | B9H01_RS09200 | lplB | sugar ABC transporter permease | 0.683 | 3.21E-08 |
|  | B9H01_RS09195 | lplC | carbohydrate ABC transporter permease | 0.695 | 4.60E-07 |
|  | B9H01_RS09190 | lplA | ABC transporter substrate-binding protein | 0.747 | 1.61E-06 |
|  | B9H01_RS09940 | fliY | amino acid ABC transporter substrate-binding protein | 0.698 | 1.50E-06 |
|  | B9H01_RS05035 | glnH | ABC transporter substrate-binding protein/permease | 1.304 | 4.52E-06 |
|  | B9H01_RS06585 | peb1B | amino acid ABC transporter permease | 1.781 | 4.10E-06 |
|  | B9H01_RS06580 | peb1B | amino acid ABC transporter permease | 2.095 | 1.44E-11 |
|  | B9H01_RS09040 | lysY | amino acid ABC transporter ATP-binding protein | 1.715 | 6.16E-09 |
|  | B9H01_RS10010 | troB | metal ABC transporter ATP-binding protein | 0.799 | 3.48E-06 |
|  | B9H01_RS01490 | ecfT | energy-coupling factor transporter transmembrane protein EcfT | 2.536 | 2.97E-41 |
|  | B9H01_RS09795 | patA | ABC transporter ATP-binding protein/permease | 1.235 | 6.46E-05 |
|  | B9H01_RS09790 | patB | ABC transporter ATP-binding protein/permease | 1.212 | 4.74E-05 |
|  | B9H01_RS02495 | artQ | amino acid ABC transporter permease | 1.488 | 0.004971 |
|  | B9H01_RS09935 | tcyB | amino acid ABC transporter permease | 0.607 | 0.004469 |
|  | B9H01_RS05040 | glnQ | amino acid ABC transporter ATP-binding protein | 1.224 | 7.90E-05 |
|  | B9H01_RS07465 | livM | branched-chain amino acid ABC transporter permease | 0.764 | 0.002623 |
|  | B9H01_RS10025 | troA | zinc ABC transporter substrate-binding protein | 0.766 | 1.76E-08 |
|  | B9H01_RS07505 | msmG | carbohydrate ABC transporter permease | 0.619 | 2.69E-06 |
| Propanoate metabolism  (0.0091) | B9H01_RS08650 | accC | acetyl-CoA carboxylase biotin carboxylase subunit | 1.327 | 9.88E-05 |
|  | B9H01_RS08660 | accB | acetyl-CoA carboxylase biotin carboxyl carrier protein | 1.285 | 0.005716 |
|  | B9H01_RS08640 | accA | acetyl-CoA carboxylase carboxyl transferase subunit alpha | 1.293 | 0.004391 |
|  | B9H01_RS07920 | atoB | acetyl-CoA C-acetyltransferase | 1.225 | 0.000261 |
| Glycerolipid metabolism  (0.029) | B9H01_RS07520 | galA | alpha-galactosidase | 0.666 | 0.000335 |
|  | B9H01_RS09415 | dhaM | PTS-dependent dihydroxyacetone kinase phosphotransferase subunit DhaM | 0.633 | 7.36E-15 |
|  | B9H01_RS09410 | dhaL | dihydroxyacetone kinase subunit L | 0.663 | 1.22E-17 |
|  | B9H01_RS09405 | dhaK | dihydroxyacetone kinase subunit DhaK | 0.642 | 4.02E-19 |
| Glycosaminoglycan degradation  (0.036) | B9H01_RS05605 | nagZ | beta-hexosamidase | 1.24 | 4.97E-05 |
|  | B9H01_RS05600 | nagZ | glycoside hydrolase family 3 protein | 1.255 | 1.61E-05 |
| Valine, leucine and isoleucine degradation  (0.036) |  |  |  |  |  |
|  | B9H01_RS03780 | ilvE | branched-chain amino acid aminotransferase | 2.194 | 2.03E-14 |
